# Bayesian Inference of Pathogen Phylogeography using the Structured Coalescent Model

**DOI:** 10.1371/journal.pcbi.1012995

**Published:** 2025-04-21

**Authors:** Ian Roberts, Richard G. Everitt, Jere Koskela, Xavier Didelot

**Affiliations:** 1 Department of Statistics, University of Warwick, Coventry, United Kingdom; 2 Zeeman Institute for Systems Biology and Infectious Disease Epidemiology Research (SBIDER), University of Warwick, Coventry, United Kingdom; 3 School of Mathematics, Statistics and Physics, Newcastle University, Newcastle, United Kingdom; 4 School of Life Sciences, University of Warwick, Coventry, United Kingdom; Ecole Normale Superieure, FRANCE

## Abstract

Over the past decade, pathogen genome sequencing has become well established as a powerful approach to study infectious disease epidemiology. In particular, when multiple genomes are available from several geographical locations, comparing them is informative about the relative size of the local pathogen populations as well as past migration rates and events between locations. The structured coalescent model has a long history of being used as the underlying process for such phylogeographic analysis. However, the computational cost of using this model does not scale well to the large number of genomes frequently analysed in pathogen genomic epidemiology studies. Several approximations of the structured coalescent model have been proposed, but their effects are difficult to predict. Here we show how the exact structured coalescent model can be used to analyse a precomputed dated phylogeny, in order to perform Bayesian inference on the past migration history, the effective population sizes in each location, and the directed migration rates from any location to another. We describe an efficient reversible jump Markov Chain Monte Carlo scheme which is implemented in a new R package StructCoalescent. We use simulations to demonstrate the scalability and correctness of our method and to compare it with existing software. We also applied our new method to several state-of-the-art datasets on the population structure of real pathogens to showcase the relevance of our method to current data scales and research questions.

## Author summary

A virus may be present in several countries, but typically most transmission events will take place within each country, with only a relatively small number of transmission events happening from one country to another. Such structure in the pathogen population has an effect on the similarity between genomes. If the geographical structure is strong then genomes collected from the same location will be more similar on average than genomes collected from different locations. Conversely, we can reverse this principle to determine what the relationships between genomes (that we observe) implies about the pathogen population structure (that we do not observe but want to learn about). Here we present a new method to perform this task. We apply it to several simulated and real sets of pathogen genomes to reveal their underlying population structure. Knowing about pathogen population structures has important consequences for understanding the evolution and epidemiology of infectious disease pathogens, and therefore to inform the public health policies that can limit their burden.

## Introduction

Genomic data is readily available for a wide range of pathogens from large online databases [1–3], and can be obtained relatively cheaply and quickly for newly acquired clinical samples [4–6]. Comparative analysis of these genomic sequences can help understand the way pathogens cause disease, spread and evolve, and this general approach is called pathogen phylodynamics [7–9]. A specific problem in phylodynamics is to try and understand the population geographical structure given genomes from several distinct locations. This approach is called pathogen phylogeography, and typically attempts to infer the relative size of the pathogen populations in each location, as well as the rates of migrations between them [10–12].

Under many forward-in-time population genetics models, including the Wright–Fisher model [13,14], the Moran model [15] and more generally the Cannings models family [16], the ancestry of a sample from an unstructured population is described by the coalescent model [17,18]. On the other hand if the population is structured following a finite island model with migrations [14], then the ancestry of samples from several locations is described by an extension of the coalescent model called the structured coalescent model. This model was first presented in the case with two locations [19], then extended to the general case [20] and its properties have subsequently been thoroughly examined [21–24]. Here we consider the structured coalescent model on a natural time scale and with leaves sampled over time [25], which enables the study of measurably evolving populations [26,27].

Inference for structured genealogies is challenging due to the mixed discrete and continuous components required to fully specify a structured genealogy, the high dimensionality of the latent genealogy, multimodality, potential for heavy tails in posteriors, complicated correlation structures, etc. Consequently, current Bayesian inference methods tend to characterise the posterior distribution using Markov chain Monte Carlo (MCMC) sampling. In general, existing methods target a large space, jointly inferring dated phylogenetic trees and geographic locations in the ancestry of the sampled lineages, although some methods can fix the dated phylogeny and sample only the ancestral locations and evolutionary parameters governing the model, usually by restricting the set of MCMC operators. Fixing the dated phylogeny significantly reduces the size of the target space, improving computational performance, but may underestimate the uncertainty in evolutionary parameter estimates and introduce biases. Existing methods fall into two broad categories, either sampling from the exact structured coalescent, or approximating the structured coalescent to integrate over parts of the migration history. Exact inference methods are computationally demanding and can fail to scale to large datasets. Approximate methods improve the scalability and can be applied to larger datasets.

By far the most widely used approach to phylogeography is discrete trait analysis (DTA) in which the geographical location is modelled as evolving independently along the branches of a genealogy in a similar manner to mutation on a genetic locus [28,29]. Whilst DTA is described by a forwards-in-time migration process for a fixed genealogy, the resulting structured genealogies can be thought of as rough approximations of structured genealogies sampled from the structured coalescent model. Less approximate models based on the structured coalescent have been proposed and implemented for example in BASTA [30] and MASCOT [31], although at the cost of being less scalable than DTA. These approximate models rely to some extent on ignoring correlation between the current state of contemporary lineages. However, applying these approximate models to data assumed to arise under the structured coalescent can lead to unpredictable biases in inferences [32]. Furthermore, integrating out parts of the migration history means that these methods cannot directly answer some simple questions of interest, such as how many migration events took place in the ancestry without additional computation. We therefore focus here on the problem of performing inference under the exact structured coalescent model.

A first MCMC scheme to perform Bayesian inference under the structured coalescent was proposed two decades ago [33], which extended MCMC operators used for inference under the unstructured coalescent [25] as well as introducing new MCMC operators which act directly on migration histories. This scheme is irreducible over the space of structured genealogies, adding or removing migration events from a migration history singly or in pairs, but suffers from poor performance with slow exploration of the target space. The authors’ implementation of the code is no longer available, however it has been reimplemented at least once before [34], and we have also reimplemented it in the current study. MultiTypeTree [34] is a package of BEAST2 [35] that extends the same set of unstructured coalescent operators [25] to the structured coalescent and introduces an additional, more sophisticated NodeRetype operator which modifies the migration history along branches of the genealogy surrounding a coalescent node. MultiTypeTree has been used as a gold standard against which approximate methods have been compared, but it is computationally demanding and cannot handle large datasets [30,32].

We propose a modular approach for phylogeographic inference using the exact structured coalescent separating the inference of a dated phylogeny and later inference of migration histories into distinct steps [36,37]. Separating the inference in this way allows our approach to better scale to the large datasets currently available in pathogen genomics. In principle the method described here could be extended to jointly infer the dated phylogeny by including existing MCMC operators for exploring the space of structured phylogenies, such as those used by MultiTypeTree. In our phylogeographic approach, a dated phylogeny is previously inferred from genomic data, either directly for example using BEAST [38] or BEAST2 [39], or by inferring dates from an undated phylogeny using methods such as LSD [40], treedater [41], TreeTime [42] or BactDating [43]. Migration histories and the evolutionary parameters governing the structured coalescent model are therefore inferred conditional on this fixed dated phylogeny and the known sampling locations of the genomes. Both previously proposed MCMC schemes [33,34] contain operators which can be used to sample migration histories for a fixed phylogeny but neither scheme is optimised for this purpose since they both considered the joint problem of inference of the phylogeny and phylogeography. We present a new MCMC scheme optimised for this purpose, which updates the migration history using as proposal distribution a localised, conditional version of DTA [28]. We apply our new method to many simulated and real datasets to demonstrate its correctness, scalability and usefulness.

## Methods

### The structured coalescent

The genealogy of a finite sample of homologous, non-recombinant individuals drawn from a larger population is widely modelled using the Kingman coalescent [17,18]. Under the Kingman coalescent, each pair of contemporary lineages is subject to a coalescent process whereby a pair of lineages coalesce at a fixed coalescent rate $\theta ={\left(N_{e}g\right)}^{-1}$, dependent on the effective population size ($N_{e}$) and generation length (*g*) of the population. A natural extension to the Kingman coalescent is to introduce population structure using an island model [14], in which each lineage is assigned to one of finitely-many demes, or subpopulations, at every time. This extension is referred to as the structured coalescent [19–24].

Under the structured coalescent, lineages are subject to three event types - sampling, coalescence and migration. A sampling event adds a new lineage to the process backwards-in-time, represented as a new leaf in the phylogenetic tree with a known isolation time and sampling deme. Coalescence events correspond to a pair of contemporary lineages in the same deme finding a common ancestor and coalescing into a single lineage backwards-in-time. Each pair of lineages currently in a deme *i* coalesce at a fixed rate $\theta_{i}$, which is equivalent to the Kingman coalescent rate for an unstructured population with effective population size equal to that of deme *i*. Migration events correspond to a single lineage moving from its current deme into a new deme, with lineages migrating from deme *i* into deme *j* (*j* ≠ *i*) at a fixed rate $\lambda_{ij}$ backwards-in-time. A structured genealogy is constructed by simultaneously tracking each of these event types backwards-in-time until the root of the entire sample is reached. Once complete, the structured genealogy consists of a phylogenetic tree *T* superimposed with a migration history *H* giving the deme membership at every point on *T*.

The structured coalescent probability density can be constructed recursively by considering the contribution from the time increments between consecutive events (sampling, coalescence or sampling). The $r^{\text{th}}$ time increment of length $\tau_{r}$ with $k_{ir}$ lineages in deme *i* (*i* = 1 , 2 , *…* , *d*) contributes a factor


Cr exp ⁡  {−∑i=1d [θikir2+ ∑j=1j≠idλijkir ]τr }. (1)

The constant $C_{r}$ depends on the type of event following the interval (backwards-in-time) and is given by


$$\begin{array}{ccc} C_{r}=\{\begin{array}{cc} 1,\; & \text{Sampling event},\\ \theta_{i},\; & \text{Coalescence event in deme}\;i,\\ \lambda_{ij},\; & \text{Migration event}\;i\to j. \end{array} & & \end{array}$$ (2)

The probability density of a structured genealogy can then be factorised as


PSC(T,H|Θ,Λ)= ∏i=1d [θici exp ⁡  {−θi ∑rkir2τr } ∏j=1j≠idλijmij exp ⁡  {−λij ∑rkirτr } ] (3)

where $c_{i}$ denotes the total number of coalescence events occurring in deme *i*, $m_{ij}$ denotes the total number of migration events from deme *i* to deme *j* backwards-in-time and $k_{ir}$ denotes the total number of lineages in deme *i* during time increment *r*.

### Discrete trait analysis

Discrete trait analysis (DTA) [28] is a widely-used phylogeographic model in which migration events are placed on a phylogenetic tree as the points of a marked Poisson point process. The position and target deme of each migration event on the phylogeny arises from a forwards-in-time migration process propagating demes from the root towards the leaves of the phylogeny. DTA is also often referred to as the mugration method [44] due to its analogue with placing mutation events on an unstructured phylogeny, or a phylogenetic continuous-time Markov chain (CTMC) model [45]. Whilst treating the migration and coalescent processes independently incurs a significantly smaller computational cost than the structured coalescent when performing likelihood-based inference, this approximation can lead to biased estimates of migration rates [30].

A structured genealogy is constructed under DTA in two phases by first sampling a dated phylogeny *T* using the Kingman coalescent, and superimposing with a migration history *H*. A migration history is obtained by sampling a deme at the root of the phylogeny and propagating forwards-in-time towards the leaves. The deme propagating through the tree evolves according to a continuous-time Markov process with transition rates given by the forwards-in-time migration rate matrix . When a coalescent event is reached, the migration process splits into two independent copies which continue evolving along each child branch. The DTA probability density of a structured genealogy with *n* samples from *d* demes is given by


PDTA(T,H|θ,F)=θn−1 exp ⁡  {−θ∑rkr2τr }⋅∏i=1d [πi ∏j=1j≠idfijmji exp ⁡  {−fij ∑rkirτr } ], (4)

where $\text{F}=(f_{ij})$ denotes the matrix of forwards-in-time migration rates, $m_{ij}$ denotes the total number of migration events from deme *i* to deme *j* *backwards-in-time*, $k_{r}=\sum_{i=1}^{d}k_{ir}$ denotes the number of contemporary lineages in time increment *r*, of length $\tau_{r}$, and $\pi_{i}$ denotes the probability of sampling the root in deme *i*. [28] originally propose sampling the root deme either from the stationary distribution of the continuous time Markov process (with forwards-in-time migration rates ) or assuming there is no long-term preference for a root deme and sampling uniformly, $\pi_{i}=\frac{1}{d}$. The forwards-in-time migration rates matrix which maintains the population flow forwards- and backwards-in-time when compared to a backwards-in-time migration process with rates matrix *Λ* and coalescent rates vector *Θ* can be computed using [46]


$$\begin{array}{ccc} f_{ij}=\frac{\theta_{i}}{\theta_{j}}\lambda_{ji}. & & \end{array}$$ (5)

Note that the parameter *θ* in Equation 4 represents the coalescent rate for the entire population considered together, and does not relate to the coalescent rates vector *Θ* in the structured coalescent.

### Bayesian inference and prior distributions

Our inferential target is the joint posterior distribution of migration histories *H*, backwards-in-time migration rate matrices *Λ* and coalescent rate vectors *Θ* conditional on a fixed phylogenetic tree *T*, genomic data *S* and sampling demes *D*. We assume throughout that the sampling deme of each sampled lineage is known and fixed, with implicit conditioning on genomic data *S* and sampling demes *D*. The joint posterior distribution can then be specified as


$$\begin{array}{ccc} p(H,\Lambda ,\Theta |T)\propto p(H,T|\Lambda ,\Theta )\cdot p(\Lambda ,\Theta ). & & \end{array}$$ (6)

The first term on the right-hand side gives the joint probability of the phylogenetic tree and migration history conditional on the coalescent rates vector *Θ* and migration rates matrix *Λ*, and is computed using the structured coalescent probability density (Equation 3). The second term gives the prior distribution on the evolutionary parameters.

The specification of prior distributions is a vital part of any Bayesian analysis, and is especially important for phylogeographic inference where a single sampling deme is recorded for each lineage [29]. Despite explicit warnings against the use of default prior distributions [47], many analyses either use default prior distributions, or fail to explicitly report alternative prior distributions [29]. In particular, MultiTypeTree [34] and the BEAST-classic implementation of DTA [28] place improper log-uniform (1 ∕ *X*) priors on both migration rates and effective population sizes. However, [29] criticise these apparently widely-used default priors as making strong, biologically unreasonable assumptions about the underlying coalescent and migration processes. Consequently, we formulate our own set of default prior distributions which are tailored to a dated phylogeny.

We use Gamma-distributed priors for our default framework, with each coalescent rate and backwards-in-time migration rate assigned an independent, and potentially unique, prior distribution. The Gamma distribution provides a flexible family of distributions with options to construct relatively diffuse, uninformative prior distributions as well as more concentrated prior distributions focussing posterior sampling into smaller regions of the target space. Gamma priors on evolutionary parameters are also conjugate with the structured coalescent likelihood (S1 Text), hence allowing exact conditional distributions to be computed and evolutionary parameter updates using Gibbs samplers.

Instead of specifying a single default prior distribution, we use an Empirical Bayes-style approach to build a default prior framework which depends on the dated phylogeny for the MCMC. Our default prior framework reflects our prior belief that migration histories of interest will contain relatively few migration events and remain fairly parsimonious. If a migration history contains many migration events, the leaf sampling demes provide little information about ancestral locations since lineages will often migrate multiple times prior to any coalescence event occurring. We begin by setting the shape parameter of each prior to *α* = 1, equivalently placing exponentially-distributed priors on each evolutionary parameter. We then match the prior expectations with the evolutionary parameters which maximise an approximation to the structured coalescent probability density (Equation 3) using the following four assumptions to remove dependence on a specific migration history:

(i) All demes have equal coalescent rates, $\theta_{i}=\theta$ for all *i* = 1 , 2 , *…* , *d*
(ii) All migration rates are equal, $\lambda_{ij}=\lambda$ for all *i* ≠ *j*
(iii) Lineages are uniformly distributed between demes at all times, $k_{ir}=\frac{k_{r}}{d}$ where $k_{ir}$ denotes the number of lineages in deme *i* during time increment *r* and $k_{r}$ denotes the number of lineages during time increment *r*
(iv) The migration history is parsimonious and contains the minimum number of required migration events M= min ⁡ HNmig(H).

Under assumptions (i)–(iv) the structured coalescent probability density satisfies


PSC(T,H|Θ,Λ)= ∏i=1d [θici exp ⁡  {−θi ∑rkir2τr } ∏j=1j≠idλijmij exp ⁡  {−λij ∑rkirτr } ](3)= ∏i=1d [θci exp ⁡  {−θ∑rkir2τr } ∏j=1j≠idλmij exp ⁡  {−λ∑rkirτr } ](i−ii)=θn−1 exp ⁡  {−θ∑i=1d ∑rkrd2τr }⋅λM exp ⁡  {−λ∑i=1d ∑j=1j≠id ∑rkrdτr }(iii−iv)≈θn−1 exp ⁡  {−θd ∑rkr2τr}⋅λM exp ⁡  {−(d−1)λL}, (7)

where $L=\sum_{r}k_{r}\tau_{r}$ is the total branch length of *T*, and we use the approximation


krd2≈1d2kr2

in the final line. This is maximised by evolutionary parameters $\left(\hat{\Theta },\hat{\Lambda }\right)$ given element-wise by


θ^i=d(n−1)∑rkr2τr,λ^ij=M(d−1)L. (8)

Matching the prior expectations with the maximisers (Equation 8) hence yields default prior distributions


θi∼Gamma (1,1d(n−1)∑rkr2τr),λij∼Gamma (1,(d−1)LM). (9)

We complete our specification of prior distributions by computing *M*, the minimum number of required migration events for phylogeny *T* using the Fitch algorithm [48]. The parameter *M* is highly correlated with the expected number of migration events in a migration history and different values of *M* may be used to vary the degree of parsimony in the prior distribution. Smaller values of *M* place stronger penalties on migration events, leading to increasing parsimony and larger values of *M* induce greater numbers of migration events.

### Markov chain Monte Carlo

We describe an irreducible Markov chain Monte Carlo (MCMC) sampling scheme to draw joint samples  ( *H* , *Θ* , *Λ* )  of migration histories and evolutionary parameters from the posterior distribution (Equation 6). Our scheme consists of two Gibbs updates for evolutionary parameters and a Metropolis–Hastings update for migration history *H*.

We construct Gibbs updates for evolutionary parameters, with one Gibbs update for the coalescent rate vector *Θ*, and a second for the backwards-in-time migration rate matrix *Λ*. Our Gamma-distributed priors are conjugate with the structured coalescent probability density for both coalescent rates and backwards-in-time migration rates, allowing conditional distributions to be evaluated analytically (Supplementary Text S1). We sample updates to evolutionary parameters elementwise from their respective Gamma-distributed conditional distributions


θi|(Λ,Θ−(i),H,T)∼Gamma (αi+ci,βi+ ∑rkir2τr )λij|(Λ−(i,j),Θ,H,T)∼Gamma (αij+mij,βij+ ∑rkirτr ), (10)

where $c_{i}$ denotes the number of coalescent events occurring in deme *i* in migration history *H*, and $m_{ij}$ denotes the number of backwards-in-time migration events from deme *i* into deme *j* in migration history *H*.

Joint samples  ( *Θ* , *Λ* , *H* )  of evolutionary parameters and migration histories *H* also require a proposal mechanism to update the migration history conditional on the current evolutionary parameters and fixed dated phylogeny. We describe a family of proposal mechanisms using a localised, conditional version of DTA to generate updates of the migration history. We select a subtree of the fixed dated phylogeny on which to update the migration history and then sample a new migration history over the subtree from a DTA model, conditioning on the demes at the points where the subtree reconnects with the remainder of the phylogeny. The conditional DTA model samples migration events under the forwards-in-time migration process described by the forwards-in-time migration rate matrix corresponding to the current evolutionary parameters  ( *Θ* , *Λ* )  using Equation 5. Sampling locally using DTA requires three steps, first selecting a subtree on which to update the migration history, then sampling a configuration of demes at any internal coalescent events of the subtree and finally sampling migration histories along each branch segment. This process relies on the fact that under DTA, migration histories along branches of a phylogeny are conditionally independent given the demes at internal coalescent events and lineage sampling demes.

**Fig 1:**
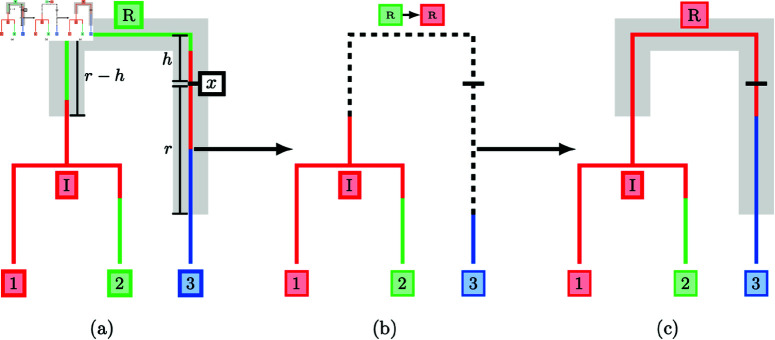
Full migration history update using a radius-based subtree selection. A subtree is selected with centre at *x* and radius *r* (a). The migration history on the subtree is erased and the deme at the internal coalescent event *R* is resampled (b). Finally, a new migration history is sampled under DTA conditional on the demes at the points where the subtree reconnects to the migration history (c).

Fig 1 illustrates our proposal for a phylogeny with three leaves. In Fig 1a, the subtree highlighted in grey is selected and the migration history for this subtree is removed. In Fig 1b, the deme at the root *R* (the only internal coalescent event) is updated conditional on the demes at the points where the subtree reconnects with the remainder of the tree before sampling new migration histories along each of the child branches of the root in Fig 1c. On the left-hand branch, the subtree reconnects in the red deme between *I* and *R* with no migration events, and on the right-hand branch the subtree reconnects in the blue deme between 3 and *R*, adding a single migration event. The completed proposal has removed two migration events (both from the red deme into the green deme backwards-in-time) and shifted the migration event from the blue deme to the red deme closer to the root of the tree. In the following sections, we highlight the mechanism used to select a subtree, update the deme at the internal coalescent event and sample each of the migration histories.

### Migration history proposal step 1: subtree selection

The choice of a subtree selection method directly affects the computational complexity of the updates. Migration history updates associated with large subtrees containing many coalescent events and a greater total branch length are generally more ambitious, potentially making large changes to the migration history, and are more computationally demanding to construct. We have settled on a radius-based approach in which we include points in the subtree based on their patristic distance from a sampled subtree centre (marked *x* in Fig 1a). We define the patristic distance between two points on a tree to be the sum of branch lengths along the unique path connecting the two points. A subtree of radius *r* is then constructed in two steps, first selecting a location *x* on the underlying tree for the subtree centre and then isolating all points at most patristic distance *r* from *x*. We sample the date of the subtree centre *x* uniformly between the dates of the root, $t_{\text{root}}$, and the most recently sampled leaf, $t_{\text{Max}}$, and then select one of the coexisting lineages at that time uniformly at random.

We use a Robbins-Monro Controlled MCMC approach [49–51] to adapt the radius *r* during the MCMC run based on the acceptance rate of migration history proposals. The adaptation begins with an initial radius $r_{0}$, a target acceptance probability $\alpha^{*}\in (0,1)$ and adaptation rate *β* ∈ ( 1 ∕ 2 , 1 ] . We typically choose an initial radius around one tenth of the total tree height, target acceptance rate $\alpha^{*}=0.234$ and adaptation rate *β* = 0 . 6. An acceptance rate of 0.234 is optimal in a range of scenarios, especially when the target distribution follows a Gaussian distribution [52]. Whilst our migration history target certainly does not follow a Gaussian distribution and we do not expect this value to be optimal for our MCMC proposal, we find that this value strikes a reasonable balance between exploring the space rapidly and the radius remaining at a reasonable size. The radius $r_{n}$ at iteration *n* is then updated on a log scale by the update rule


log ⁡ rn= log ⁡ rn−1+n−β(α(H′|Hn−1)−α∗), (11)

where $\alpha (H^{\prime }|H_{n-1})$ denotes the acceptance probability of a migration history $H^{\prime }$ sampled from previous state $H_{n-1}$.

### Migration history proposal step 2: coalescent node sampling

After selecting a subtree, we update the deme at each coalescent event contained within the subtree, conditioning on the current deme at each point where the subtree reconnects to the remainder of the migration history. These fixed demes correspond to the leaves of the subtree, and the root of the subtree provided the subtree root is not the overall tree root. We use belief propagation [53,54] to compute the conditional distributions at each coalescent node using transition rates derived from the forwards-in-time migration rate matrix corresponding to the current evolutionary parameters  ( *Θ* , *Λ* ) . In place of computing a full conditional distribution at each coalescent event, we draw samples from the joint distribution of demes using a backward filtering-forward sampling approach similar to the forward-backward algorithm for Hidden Markov Models [55]. We describe this approach applied to a tree containing only fixed leaf demes, however this can be extended to subtrees where the root deme is also fixed by passing a single message from the subtree root to its children.

The backward filtering pass begins at the leaves of the subtree and passes messages towards the root along all parent edges, following Felsenstein’s pruning algorithm [56]. Each message consists of a vector of unnormalised probabilities that the parent node falls into each deme conditional on the fixed demes of all descendant subtree leaves. Formally, a node *i* passes a message $\mu_{ij}$ to its parent *j* consisting of elements


$$\begin{array}{ccc} \mu_{ij}(d_{j})=\underset{d_{i}}{\sum }\left(P_{\left\langle i,j\right\rangle }(d_{i}|d_{j})\underset{k\in C(i)}{\prod }\mu_{ki}(d_{i})\right) & & \end{array}$$ (12)

where *C* ( *i* )  denotes the set of child nodes of *i*, $d_{*}$ denotes the deme at node * and $P_{\left\langle i,j\right\rangle }(y|x)$ denotes the transition probability of migrating forward-in-time from deme *x* to deme *y* along edge  ⟨ *i* , *j* ⟩ . A leaf node *i* with a fixed deme has no incoming messages and simply passes the message


$$\begin{array}{ccc} \mu_{ij}(d_{j})=P_{\left\langle i,j\right\rangle }(d_{i}|d_{j}) & & \end{array}$$ (13)

to its parent *j*. All incoming messages to a node must be determined before a message can be passed onwards, which is achieved by computing messages in time order, beginning at the most recent leaf of the subtree and ending at the eldest child of the subtree root. Once all messages have been passed to the root of the tree, the conditional deme distribution at the root is computed by taking the product of all incoming messages.

**Fig 2:**
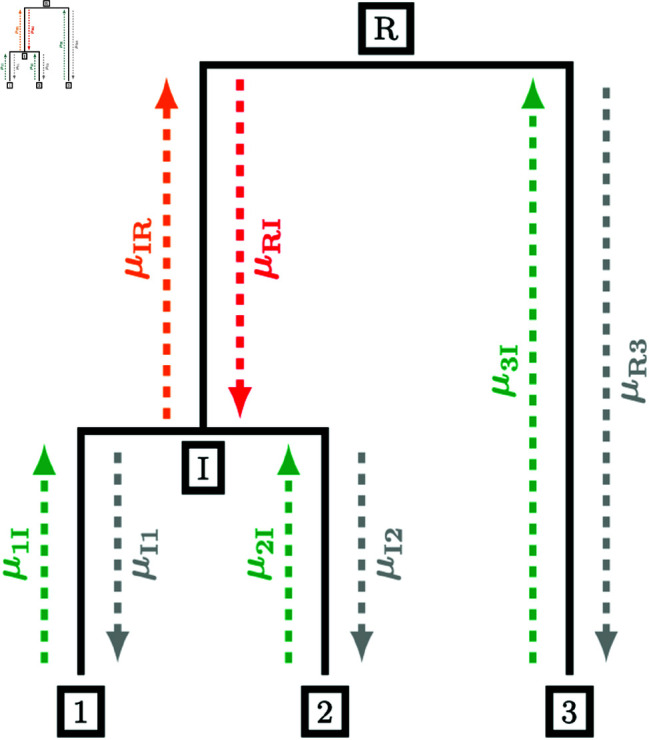
Backward filtering-forward sampling messages for a tree with 3 leaves. Messages are computed in the order green-orange-red, and messages in grey could be computed but are unnecessary to sample a new configuration of demes at nodes *I* and *R.*

We begin the forward sampling pass by sampling from the conditional distribution at the root. The root is then treated as fully determined and passes messages to its child nodes using Equation 13. The child nodes now have all incoming messages fully determined and the conditional deme distribution can be computed by taking a product of incoming messages. This process is repeated iteratively until a new deme has been sampled at every coalescent event in the tree. Fig 2 illustrates the messages required for a full pass of the backward filtering-forward sampling algorithm. The demes are computed in the order green-orange-red and the deme distributions at *R* and *I* are sampled from


$$\begin{array}{rcll} P_{R}(d_{R}) & \propto & \mu_{3I}(d_{R})\cdot \mu_{IR}(d_{R}), & \\ P_{I}(d_{I}) & \propto & \mu_{1I}(d_{I})\mu_{2I}(d_{I})\mu_{RI}(d_{I}) & \end{array}$$

respectively. The messages in grey would be given by a point mass as the transition probability from the determined parent to the determined child along the edge but are unnecessary to sample a deme configuration.

### Migration history proposal step 3: migration history sampling

A migration history proposal is completed by sampling the migration history along each branch in the subtree conditional on the demes at the subtree leaves, root and coalescent events. Under the DTA model, the migration history along each branch is a conditionally independent realisation of the forwards-in-time Markov process described by the forwards-in-time migration rate matrix and these fixed demes. We refer to these realisations of Markov processes conditioned on both their initial and final states as Poisson Bridges, and there are a number of ways to sample realisations [57]. We adopt the uniformization scheme in which an auxiliary Markov process is constructed with events occurring faster than in the original migration process whilst also permitting self-migrations, where a lineage migrates back into its current deme. The total number of events occurring in the auxiliary Markov process is then sampled and the deme at each jump time of the auxiliary process is sampled from a discrete-time Markov chain whilst conditioning on the deme at both ends of the branch migration history. Finally, a migration history is constructed by thinning the events and removing self-migrations in the auxiliary process.

The acceptance probability of a migration history update $H_{1}$ sampled from current state $H_{0}$ is computed with the standard Metropolis–Hastings acceptance ratio


$$\begin{array}{ccc} \alpha (H_{1}|H_{0})=\min\left(1,\frac{P(H_{1}|T,\Theta ,\Lambda )}{P(H_{0}|T,\Theta ,\Lambda )}\cdot \frac{q_{\Theta ,\Lambda }(H_{0}|H_{1})}{q_{\Theta ,\Lambda }(H_{1}|H_{0})}\right). & & \end{array}$$ (14)

The terms in the first ratio of Equation 14 are given by the structured coalescent probability density (Equation 3) evaluated for the entire structured phylogeny, noting that


$$\begin{array}{ccc} P(H|T,\Theta ,\Lambda )=\frac{P(T,H|\Theta ,\Lambda )}{P(T|\Theta ,\Lambda )}\propto P_{\text{SC}}(T,H|\Theta ,\Lambda ). & & \end{array}$$ (15)

The transition kernel $q_{\Theta ,\Lambda }(H_{1}|H_{0})$ in Equation 14 can be decomposed as


$$\begin{array}{cccc} q_{\Theta ,\Lambda }(H_{1}|H_{0}) & =P(S)\cdot P_{\text{DTA}}(H_{1}^{\left(S\right)}|H_{0}^{-(S)},\Theta ,\Lambda ,T)\cdot P(H_{0}^{-(S)}|\Theta ,\Lambda ,T) & & \\ & \propto P_{\text{DTA}}(H_{1}^{\left(S\right)}|H_{0}^{-(S)},\Theta ,\Lambda ,T), & \end{array}$$ (16)

where *S* denotes the selected subtree, $H_{1}^{\left(S\right)}$ denotes the migration history of $H_{1}$ associated with *S*, and $H_{0}^{-(S)}=H_{1}^{-(S)}$ denotes the migration history of $H_{0}$ and $H_{1}$ associated with the complement of *S* respectively. This decomposition relies on the conditional independence of $H^{\left(S\right)}$ and $H^{-(S)}$ given the demes at the points reconnecting the migration history, and permits the transition kernel to evaluate the DTA probability density (Equation 4) locally on subtree *S* rather than the entire phylogeny *T*. Note that we evaluate the DTA probability density using the forwards-in-time migration process corresponding to the current evolutionary parameters  ( *Θ* , *Λ* )  using Equation 5.

### Implementation

The MCMC method described above is implemented into a stand-alone R package StructCoalescent available at https://github.com/IanPRoberts/StructCoalescent under a GPL-3 license. R was selected for ease of coding and debugging as well as to make use of an existing ecosystem for handling phylogenetic trees. We placed a greater focus on a correct implementation of the method than computational performance although multiple commonly-used functions, including the structured coalescent and DTA probability densities, are implemented in C++ using Rcpp-1.0.11 [58]. Phylogenetic trees were converted between BEAST metacommented nexus files and internal data structures using treeio-1.27.1 [59,60], and phylogenetic trees were plotted using ape-5.7.1 [61]. We include a function to sample endpoint-conditioned continuous time Markov processes adapted from ECctmc [62] due to the removal of this package from CRAN in January 2024. MCMC chains were run using the University of Warwick HPC facilities which place a 48-hour wallclock limit on individual runs. We opted to terminate jobs at the wallclock limit to permit repeatable analyses with consistent random number seeds for each run.

## Results

### Application to a single simulated structured phylogeny

We begin by illustrating the use of our Bayesian methodology applied to a single structured phylogeny drawn from the structured coalescent probability density (Equation 3). The phylogeny was sampled using MASTER [63], and consisted of 1000 leaves from 6 demes with coalescent rates sampled independently from an exponential distribution with rate 0.5 and backwards-in-time migration rates sampled independently from an exponential distribution with rate 20. Each leaf was assigned an isolation time sampled uniformly across a 50-unit time period with a sampling deme selected uniformly from the 6 demes. We ran six independent MCMC chains for this phylogeny using our default priors (Equation 9) with evolutionary parameters initialised at their respective prior means and initial migration histories sampled from the DTA probability density (Equation 4) such that each run used a different deme at the root. All runs were terminated following 48 hours of run time and completed between 2,400,000 and 2,510,000 iterations.

Our MCMC samples mixed well over the target space with low autocorrelation in evolutionary parameter estimates and consistent regions of the target space explored in each run. Effective sample size (ESS) estimates for each evolutionary parameter exceeded 500, with mean ESS values in excess of 700, indicating relatively low autocorrelation between consecutive thinned parameter values. We also compute joint ESS [64] to assess higher-dimensional cross-correlations between groups of parameters and found estimates exceeding 1,100 for every MCMC sample. Convergence to consistent regions of the target space was assessed with the Gelman–Rubin $\hat{R}$ statistics [65], with every per-parameter $\hat{R}$ statistic below 1.002 and multivariate $\hat{R}$ of 1.008. Whilst we are not using an explicit cutoff, such low $\hat{R}$ values provide no concern about a lack of convergence. Full results for ESS and $\hat{R}$ estimates of this analysis are available in S1 and S2 Tables.

**Fig 3:**
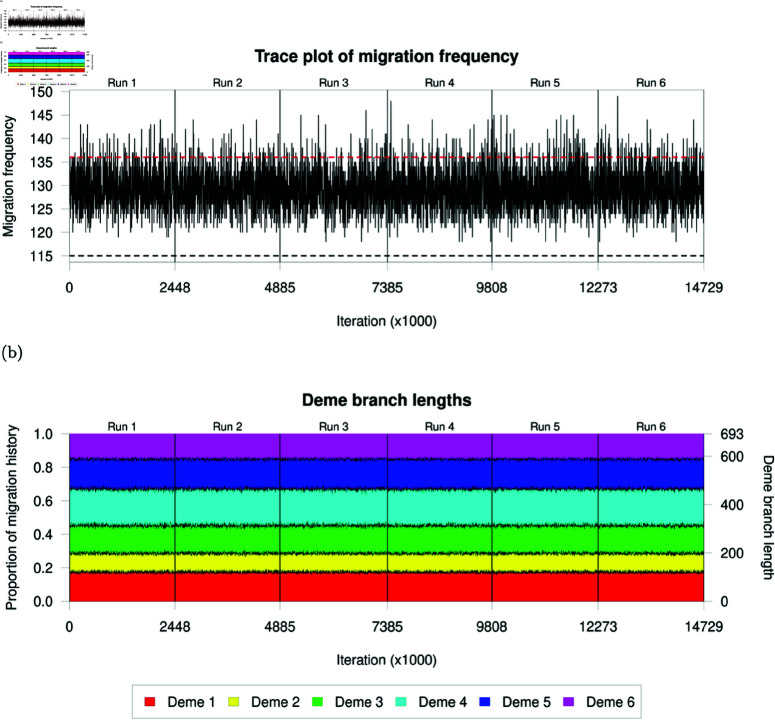
Assessment of MCMC convergence and mixing. (a) Trace plot of the total migration count. The red dashed line indicates the number of migration events in the simulated phylogeny and the black dashed line indicates the minimum number of required migration events for a consistent migration history. (b) Stacked trace plot of the proportion of the migration history falling into each deme across the six MCMC chains.

Convergence of evolutionary parameters alone is insufficient to conclude that MCMC chains have explored the target space effectively [66]. We cannot directly use moment-based convergence diagnostics on samples of migration histories due to their mixed discrete and continuous components, and instead apply diagnostics to numeric summary statistics of samples of migration histories. In particular, we considered the migration frequency and total branch length in a deme to summarise the quantity of migration events and how they are distributed across the phylogeny. Fig 3 shows trace plots of these summary statistics for each of the six MCMC chains. Migration frequencies change frequently whilst remaining in a relatively consistent region of the posterior space in every MCMC sample (Fig 3a). We infer a median of 129 migration events (95% CI [122,139]), a little lower than the 137 migration events in the simulated phylogeny which we treat as a ground truth, but we maintain reasonable coverage of the ‘true’ simulation value. We see even more consistency in the stacked trace plots of branch lengths assigned to each deme (Fig 3b) with every run observing similar proportions of the migration history in each deme whilst maintaining reasonable variability at the boundaries between demes. Overall, these two sets of trace plots provide no reason to believe that we are failing to explore the space of migration histories effectively.

**Fig 4:**
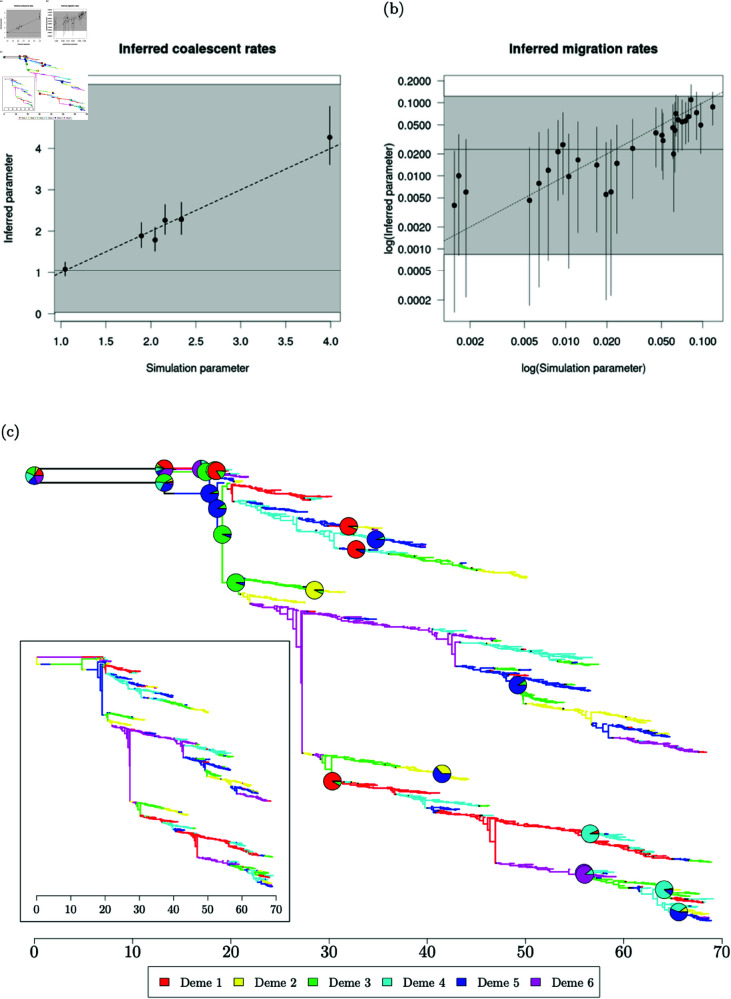
60% consensus migration history and 95% posterior credible intervals of evolutionary parameters for MCMC samples on a single simulated structured phylogeny. Samples are aggregated over all six chains prior to computations. (a) 95% posterior credible intervals for coalescent rate estimates. (b) 95% posterior credible intervals for backwards-in-time migration rate estimates. (c) 60% consensus migration history. Inset: Structured phylogenetic tree simulated using MASTER.

Once we are satisfied that our MCMC chains have converged over both evolutionary parameters and migration histories, we can move to assess the quality of posterior samples. We treat the evolutionary parameters used in the MASTER simulation as the ground truth and study the inferred 95% posterior credible intervals of evolutionary parameters. Simulation parameters are recovered well across the full range of values (Fig 4a and 4b). Inferred coalescent rates tend to fall close to the simulation parameter in every deme with high correlation between the posterior median and simulation parameter across the full range of simulation values. We have fairly precise estimates with relatively narrow credible intervals and little systematic bias with one posterior median coalescent rate slightly underestimating the simulation parameter and one slightly overestimating the parameter. Migration rates are inferred less precisely than coalescent rates with relatively broad credible intervals and lower correlation with the posterior median, especially for simulation parameters below 0.005. This loss of precision in estimates of small migration rates has been previously noted [31] and corresponds to migration events which are rarely, if ever, observed in a sampled migration history. When no migration events are observed, the conditional posterior distributions used for Gibbs sampling are updated only by the total branch length in the source deme of the migration event. This is bounded between 0 and the total branch length of the phylogeny, although in practice we expect every deme to feature in the migration history and consequently approach this upper bound infrequently.

We summarise MCMC samples of migration histories using a 60% majority consensus migration history (Fig 4c). Each point in a majority consensus history is assigned to a deme if and only if at least a proportion *p* ∈ ( 0 . 5 , 1 ]  of sampled migration histories observe the same deme at that point. Points with insufficient consensus are assigned to a ‘null deme’ which we typically plot in black. Near the leaves, we know sampling demes with certainty and consequently there is high consensus about demes along branches close to the leaves with most of the migration history fully coloured. As we move further from the leaves, uncertainty typically increases and we find an increasing proportion of the migration history assigned to the null deme. A similar observation was made for analyses using BASTA [30], with posterior probability of the modal deme decreasing further from the leaves, although we recover greater resolution by using a consensus method for the exact structured coalescent by considering the precise location and type of migration events. We identify the greatest uncertainty near the root with both child branches fully assigned to a null deme. The strong heterochronicity of leaf sampling and clustering of leaves within the same deme lead to fairly parsimonious migration history samples, and we identify much of the tree to be identical to the original simulation (Fig 4c inset). We also superimpose pie charts onto coalescent events giving the posterior distribution of demes at those events, omitting pie charts with greater than 95% consensus. Despite our simulation finding the root in deme 2, the pie chart at the root shows that our posterior samples rarely used deme 2 at the root. In the simulated migration history, the root was in deme 2. We do not expect to recover every feature of the simulated migration history, and in this case it appears that the state of the simulation was relatively unlikely under our target posterior distribution.

### Application to multiple simulated structured phylogenies

We completed a large-scale simulation study varying the number of demes and degree of heterochronicity. Five structured phylogenies, each with 1000 leaves were simulated using MASTER for each number of demes between 2 and 10 and four degrees of heterochronicity: lineage isolation dates sampled across a 0-unit time period (homochronous), 10-unit time period (mildly heterochronous), 30-unit time period (moderately heterochronous) or a 50-unit time period (strongly heterochronous). Coalescent rates used in simulations were sampled independently from an exponential distribution with rate 0.5 and backwards-in-time migration rates were sampled independently from an exponential distribution with rate 20. Two 48-hour MCMC chains were run for each phylogeny using our default prior distributions which allowed us to complete a basic convergence analysis on all 180 phylogenies.

Unlike existing methods for exact inference using the structured coalescent, we incur little penalty in per-iteration computation times with relatively stable sample sizes for all numbers of demes (S3 Table). The lack of computational penalty is caused by our adaptive scheme (Equation 11) which tends to converge to smaller subtree radii as the deme count increases. A smaller proposal radius typically reduces the number of coalescent events contained in a subtree and also reduces the branch length over which Poisson bridges must be sampled. We identify particularly slow performance for the phylogenies with 2 demes, which is also related to the adaptive scheme. Some of these phylogenies had typical acceptance probabilities in excess of our target $\alpha^{*}=0.234$ even when updating the entire migration history at every iteration, causing the proposal radius to increase without bound and reducing to an independence sampler. Independence sampler performance could be improved by skipping the subtree selection step, but we find these cases to be rare with more than 2 demes. A greater penalty arises from increasing the degree of heterochronicity, although this is likely caused by increasing the total size of the phylogenies with total branch lengths increasing from a mean of 176 units for homochronous phylogenies to 876 for phylogenies with strong heterochronicity.

We check for signs of poor convergence in our MCMC chains using multivariate $\hat{R}$ and ESS applied to each pair of samples (S3 Table). We identify pairs of samples with multivariate $\hat{R}$ exceeding 1.2 as having failed to converge, hence excluding 33 out of 180 pairs of samples. Other analyses using BASTA and MASCOT have reported similar rates of runs determined to have failed to converge using an alternative criteria of all evolutionary parameter ESS values exceeding 200 [45]. A minimum ESS criteria appears to be slightly milder, excluding only 6 pairs of samples from our analysis, although all 6 runs excluded on the ESS criteria were already excluded from our $\hat{R}$ criteria. $\hat{R}$ values increased with both number of demes and degree of heterochronicity, with both increases indicating greater difficulty in sampling. Increasing the number of demes leads to a quadratic increase in the number of inferred parameters and a more diverse space of migration histories which can make sampling more challenging. Further, poor convergence in a single parameter is often sufficient to increase the multivariate $\hat{R}$ statistic to indicate poor convergence overall. The increase in $\hat{R}$ statistics associated with increasing heterochronicity may again be correlated with increases in the total branch length of the considered phylogenies. We appear to be approaching the performance limit of our method with 48 hours of run time and phylogenies consisting of 1000 samples around 7 demes with light heterochronicity (10-units leaf sampling) although we maintain reasonable performance with homochronous sampling up to 10 demes. Further computation time applied to these phylogenies could improve the quality of posterior samples with substantially larger sample sizes, but smaller phylogenies with fewer sampled lineages (or fewer demes) would also permit greater numbers of demes to be considered with similar run times. Considering only the 147 run pairs which we deemed to have converged (multivariate $\hat{R}\leq 1.2$), we obtained ESS exceeding 230 for every individual parameter and mean joint ESS values exceeding 1,000 and frequently exceeding 2,000 (S3 Table). This indicates very low autocorrelation in MCMC samples and joint ESS around 1,000 should be sufficient to characterise moderate quantiles of the posterior distribution although may still be insufficient for a full characterisation of events far into the tails of the distributions.

**Fig 5:**
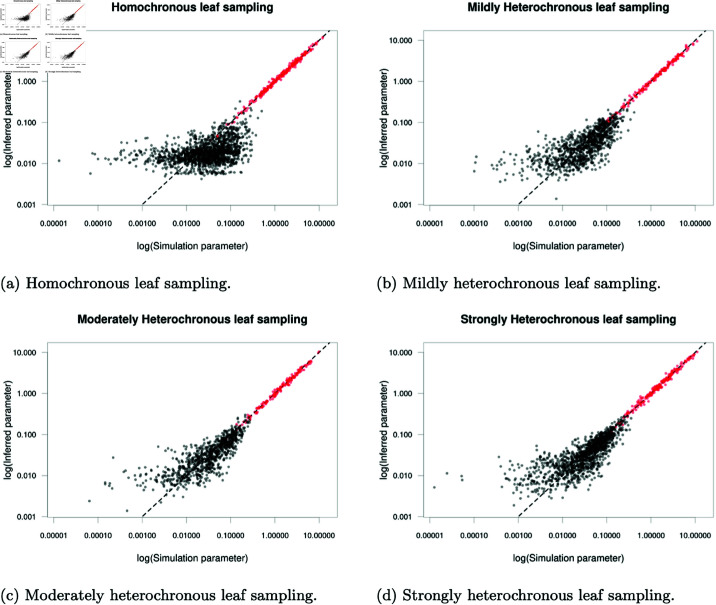
Posterior median of inferred evolutionary parameters plotted against the known simulation parameters. Inferred coalescent rates are plotted in red and inferred migration rates are plotted in black.

We now focus on assessing the quality of evolutionary parameter samples, in particular the capacity to recover the known rates used in simulations. Fig 5 shows the inferred posterior median of evolutionary parameters against the known simulation parameters. Both MCMC chains for each phylogeny were combined before computing posterior medians and we excluded pairs of samples with $\hat{R}$ exceeding 1.2. Coalescent rates (plotted in red) are inferred accurately across the full range of tested values, with small deviations from the simulation parameter. Migration rates (plotted in black) are also inferred accurately for larger values, although with lower precision than coalescent rates. Smaller migration rates are inferred less accurately with inferred values flattening out around a posterior median of 0.01, again due to infrequent observation of migration events associated with the smallest migration rates.

We also assess the quality of inferred parameters numerically using summary statistics computed by again combining pairs of MCMC chains on the same phylogeny and omitting pairs of chains with multivariate $\hat{R}$ exceeding 1.2 (Table 1). The coverage statistic is the proportion of 95% posterior credible intervals containing the simulation parameter. Theoretically, a sample from the posterior distribution should have coverage of 0.95 provided the observed data is generated using parameters sampled from their prior, with values in excess of 0.95 regarded as being overly conservative. In practice, we do not sample simulation parameters from their respective priors and consequently do not expect to recover the theoretical maximum coverage of 0.95. We identify a mean coverage of 0.896 over the 294 accepted MCMC runs, slightly below the theoretical maximum value of 0.95, but we are satisfied that we have reasonable power to recover the true underlying parameters and are satisfied with this mean coverage. The lowest coverage reliably occurs for the homochronous phylogenies (top row in Table 1) with a mean coverage of 0.847 but all other degrees of heterochronicity recover the simulation parameters more frequently. This indicates that some heterochronicity within the sampled leaves provides a stronger signal to inform the inference of evolutionary parameters. We find extremely high correlation between the posterior medians and the known simulation parameters, with all reported correlation values exceeding 0.95, indicating that estimates of the simulation parameter are close to the simulation values. Whilst this may appear inconsistent with the flattening of values observed in the plots of posterior median against simulation values (Fig 5), the observed deviations occur only for the smallest migration rates with small absolute deviations which have little effect on the correlation overall.

**Table 1:** Coverage, correlation, relative bias and relative root mean squared error (RMSE) for inferred evolutionary parameters, separated by number of demes and degree of heterochronicity in leaf sampling. Reported values are the mean taken over pairs of MCMC chains with multivariate $\hat{R}$ below 1.2.

	2 demes	3 demes	4 demes	5 demes	6 demes	7 demes	8 demes	9 demes	10 demes
**Coverage**									
Homochronous	0.800	0.867	0.838	0.864	0.935	0.792	0.872	0.840	0.812
Mild Heterochronicity	0.900	0.956	0.975	0.910	0.944	0.918	0.945	0.922	0.917
Moderate Heterochronicity	0.900	0.926	0.938	0.912	0.938	0.939	0.938	0.914	0.920
Strong Heterochronicity	0.900	0.911	0.963	0.952	0.944	0.939	0.958	0.933	0.910
**Posterior median correlation**									
Homochronous	0.977	0.999	0.998	0.997	0.997	0.996	0.992	0.993	0.996
Mild Heterochronicity	0.983	0.996	0.999	0.998	0.997	0.994	0.998	0.996	0.994
Moderate Heterochronicity	1.000	1.000	0.998	0.998	0.998	0.996	0.996	0.995	0.994
Strong Heterochronicity	1.000	0.999	0.998	0.998	0.998	0.996	0.996	0.996	0.992
**Relative bias**									
Homochronous	–0.085	0.558	–0.077	–0.198	0.038	–0.303	–0.128	–0.145	–0.219
Mild Heterochronicity	0.039	0.116	0.082	–0.042	0.003	0.008	0.117	–0.046	–0.020
Moderate Heterochronicity	0.068	0.048	0.059	–0.019	0.029	0.045	0.048	0.053	0.082
Strong Heterochronicity	–0.004	–0.027	0.054	0.119	0.011	0.019	0.068	0.039	0.036
**Relative RMSE**									
Homochronous	0.499	1.329	0.812	0.706	0.887	0.731	0.875	0.875	0.867
Mild Heterochronicity	0.263	0.519	0.575	0.553	0.659	0.729	0.732	0.700	0.777
Moderate Heterochronicity	0.326	0.377	0.486	0.468	0.560	0.640	0.682	0.754	0.771
Strong Heterochronicity	0.237	0.267	0.418	0.535	0.522	0.598	0.641	0.691	0.737

Due to the huge range in sizes of different evolutionary parameters, with the smallest migration rates around 10^–5^ and the largest coalescent rates greater than 10, we assess the precision of our posterior samples using relative bias and relative root mean square error (RMSE) to normalise our posterior samples (Table 1). Reported relative bias and RMSE estimates are reported only for simulation parameters greater than 0.01 due to the lack of statistical power in estimating small migration rates. We compute the relative bias and RMSE of a sample ${\left(\varphi_{n}\right)}_{n=1}^{N}$ with true value *φ* as


$$\text{Bias}=\frac{1}{N}\overset{N}{\underset{n=1}{\sum }}\frac{\varphi_{n}-\varphi }{\varphi },\;\text{RMSE}=\sqrt{\frac{1}{N}\overset{N}{\underset{n=1}{\sum }}{\left(\frac{\varphi_{n}-\varphi }{\varphi }\right)}^{2}}.$$

Overall, we do not find a systematic bias in our inferences with a mixture of positive and negative relative biases in our parameter estimates. Relative biases are also extremely small with few large deviations indicating accurate recovery of the simulation parameter. RMSE values are also reasonably small, indicating reasonably narrow credible intervals and fairly precise estimates of simulation parameters.

### Evolution of *Staphylococcus aureus* ST239

*Staphylococcus aureus* ST239 was one of the first bacterial lineages to have its geographical structure investigated using whole genome sequencing [67]. A phylogenetic tree from this pioneering study is available on PathogenWatch [68], consisting of 58 genomes sampled between 1982 and 2007 across 5 continents: Europe, South America, North America, Asia and Australia. This phylogeny was dated using BactDating [43] under the additive relaxed clock model [69] (S1 Fig). We take this dated phylogeny alongside the leaf sampling dates and locations as input and use our methodology on this dataset to resolve the phylogeography of ST239.

In the absence of specialist knowledge about the likely migration and coalescent rates of ST239, we use our default priors for this analysis (Equation 9), in this case $\theta_{i}∼\text{Gamma}(1,10.79)\text{ and }\lambda_{ij}∼\text{Gamma}(1,218.79)$. We ran five MCMC chains using these prior distributions, each terminated at 48 hours of run time, with each chain completing between 3,100,000 and 3,300,000 iterations. Initial migration histories were sampled from the DTA probability density (Equation 4) such that each initial migration history sampled the root in a different deme and initial coalescent and backwards-in-time migration rates were set to their respective prior means. We identify little autocorrelation within MCMC samples with all per-parameter ESS values exceeding 300 and joint ESS exceeding 1,200 in every sample. There is also no evidence of a lack of convergence with per-parameter $\hat{R}$ estimates below 1.005 and a multivariate $\hat{R}$ estimate of 1.023 on the basis of the five independent MCMC chains. Full results for ESS and $\hat{R}$ are available in S4 and S5 Tables, respectively. There is no evidence to suggest that samples of migration histories have failed to converge, with reasonable exploration of migration frequencies and branch lengths in each deme (S2 Fig). The range of migration counts is relatively consistent across the five MCMC runs, with some migration histories reaching maximum parsimony at a migration count of 8 and a maximum of 26 migration events observed in any run. Similarly, we find relatively consistent proportions of each deme explored whilst maintaining reasonable variability at the boundary between demes despite initialising each MCMC sample at distinct migration histories.

**Fig 6:**
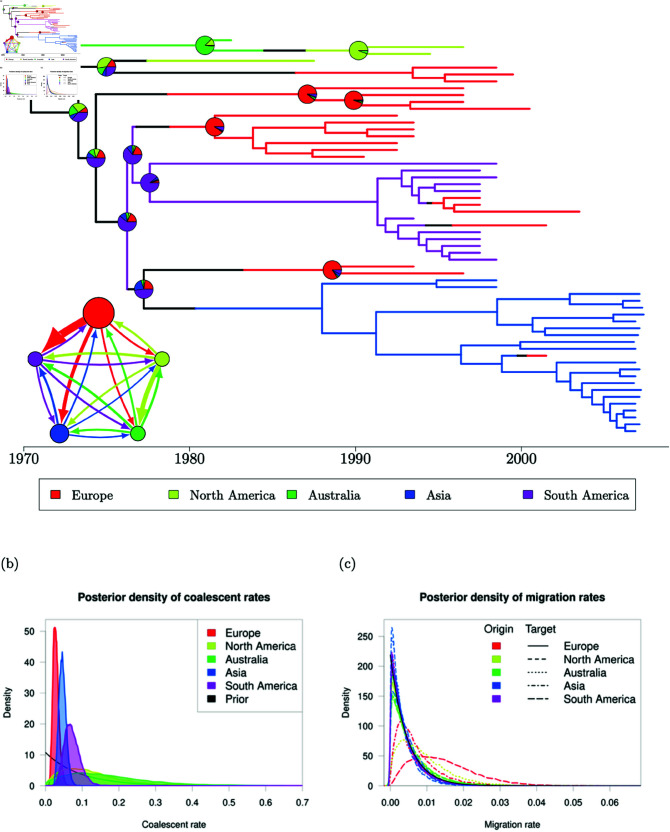
60% consensus migration history and kernel density estimates of the posterior density of evolutionary parameters for the *S. aureus* ST239 analysis. (a) 60% consensus migration history. Inset: migration model with 5 demes. The radius of each circle is proportional to the median effective population size (inverse coalescent rate) for that deme and the width of an arrow connecting two demes is proportional to the median backwards-in-time migration rate between that pair of demes. (b) Kernel density estimates of the posterior density of coalescent rates. (c) Kernel density estimates of the posterior density of backwards-in-time migration rates.

Our 60% majority consensus migration history (Fig 6a) is relatively parsimonious with most lineages coalescing with other lineages in the same deme before migrating away. We infer a posterior median of 13 migration events in a migration history (95% CI [9–18]), compared to a maximum parsimony migration history which requires 8 migration events. The original analysis [67] did not attempt to infer migration histories but noted that there was strong geographical clustering of the ST239 global population. Our parsimonious consensus migration history agrees well with this conclusion. The posterior probability for the location at the root was 44% in the Australian deme, 26% in the South American deme, 19% in the North American deme and the remaining 11% split almost evenly between Europe and Asia. The deme sampled at the root is clearly influenced by the proximity of the only Australian sample (top-most lineage) to the root. The scarcity of Australian samples provides little information about the Australian deme and we only observe the Australian lineage for a short time near the root.

We also have access to posterior samples of coalescent and migration rates. Fig 6b and 6c show kernel density estimates of the coalescent rates and backwards-in-time migration rates respectively. Assuming that the typical generation length of an individual remains consistent between continents and throughout time, the coalescent rate in a deme is inversely proportional to the effective population size. We observe sharp peaks in the density of coalescent rates in Europe, Asia and South America, with the respective modes indicating the smallest coalescent rates in Europe and largest in South America. From this, we can deduce that the largest ST239 population is found in Europe, with smaller population sizes in Asia and South America respectively. Despite the large population size in Europe, we have low probability of observing the European deme before 1970 in the consensus migration history, and we tend to require multiple importations of ST239 into Europe to account for its later appearance and widespread sampling. The coalescent rate estimates for the Australian and North American demes are more diffuse, with less concentrated peaks in the posterior density, although both empirical posteriors have been updated away from the prior distribution (shown in black). The more diffuse posterior for these parameters is likely related to the relative scarcity of samples from these demes - only 1 sample is available from Australia and 3 samples from North America, compared to 11 South American samples, 21 European samples and 22 Asian samples. The empirical posteriors for the migration rates are less well separated, with many posteriors centred close to 0. S3 Fig shows the empirical posteriors separated by the origin deme of the migration rate. We find that many of the empirical posteriors are close to the prior distribution (shown in black), indicating that the phylogeny provides little information to update parameters away from the prior distribution for small parameter values. The greatest inferred migration rates are the migration rates from Europe into South America and Asia, and the migration rate from North America into Australia (Fig 6a, inset), which are well separated from the prior distribution. We can interpret these rates as infections spreading forwards-in-time from South America and Asia into Europe and from Australia into North America. The posterior distributions for the migration rate from Europe into South America is broader with a less-defined peak and consequently there is higher uncertainty in the precise value of this parameter than most of the other migration rates.

### Comparison with previous software

We analysed this ST239 phylogeny using MultiTypeTree-8.1.0 [34] in BEAST2 with initial structured phylogenies matching those used in our original analysis. The underlying phylogeny was kept fixed throughout each run by restricting the operator set to the NodeRetype operator which can update the migration history, and two ScaleOperators to update evolutionary parameters whilst maintaining the underlying phylogeny. We used mathematically-equivalent priors to our analysis consisting of an inverse gamma prior on effective population sizes (inverse coalescent rates) and a gamma-distributed prior on backwards-in-time migration rates with the shape and rate of each prior matching our default prior distributions. Unfortunately, we found clear evidence of a lack of convergence in every MCMC sample with divergence in both the posterior density and migration counts (A and B Fig). Log-posterior densities increased rapidly from initial values around -300 to positive values in excess of 5000 (posterior density in excess of $e^{5000}\approx 10^{2171}$). This divergence in posterior density is likely to have been driven by the corresponding increase in migration counts, with over 1000 migration events commonly placed in a migration history. To eliminate the possibility of errors arising from our choice of prior distributions, we also reran MultiTypeTree using diffuse lognormal(0,4) priors on both effective population sizes and migration rates. MCMC samples drawn using different prior distributions will also have different posterior distributions and are hence incomparable, but obtaining well-converged MCMC samples using lognormal priors would indicate an error in our original prior specification. We identified a similar divergence to high log posterior densities and high migration frequencies (C and D Fig), although the divergence is less pronounced than the initial analysis with Gamma-distributed priors. We have been unable to run MultiTypeTree stably for this phylogeny using two different families of prior distributions on evolutionary parameters. We are unsure what might be causing this instability but note again that MultiTypeTree was not designed with inference on a fixed phylogeny in mind, and is hence not optimised for this task [34]. We also analysed this ST239 phylogeny using MASCOT-3.0.7 [31] in BEAST2 with five MCMC chains using mathematically-equivalent prior distributions on effective population sizes and backwards-in-time migration rates. Initial migration rates and effective population sizes were set to their respective prior means for the analysis, but unlike analyses with StructCoalescent or MultiTypeTree, MASCOT does not require an initial migration history and consequently every run was initialised at the same unstructured dated phylogeny. In practice, we performed fixed phylogeny analyses using MASCOT by restricting to two MCMC operators - one which updated the vector of effective population sizes, and a second which updated the backwards-in-time migration rates, both whilst integrating over all possible migration histories under the MASCOT model. Despite integrating over migration histories during the MCMC run, MASCOT also computes full structured phylogenies including explicit migration event locations and types which permit us to also assess mixing over sampled migration histories.

As an approximation to the structured coalescent, MASCOT is designed to gain in computational efficiency by ignoring correlations between states of contemporary lineages. Consequently, MASCOT runs significantly faster than StructCoalescent, completing over 500 million iterations in every run compared with around 3 million iterations using StructCoalescent. Due to the significantly larger sample sizes, ESS estimates are substantially higher, although we note that MASCOT does also return a slightly higher per-iteration effective sample rate with a mean of 0.66 effective samples per 1,000 iterations, slightly higher than the mean of 0.31 effective samples per 1,000 iterations from StructCoalescent. We note that the number of completed iterations and evolutionary parameter ESS obtained using StructCoalescent could be inflated by attempting the computationally-cheaper evolutionary parameter updates more frequently. In the present analysis, migration history updates were attempted 1,000 times more often than either evolutionary parameter update whereas MASCOT integrates over migration histories for parameter combinations at every iteration. We also find comparable results for convergence of evolutionary parameters to our StructCoalescent analysis, with a maximum per-parameter $\hat{R}$ statistic of 1.001 and a multivariate $\hat{R}$ statistic of 1.0016. Full ESS and $\hat{R}$ results are available in S6 and S7 Tables.

Whilst MASCOT does not focus on migration history inference, a single migration history is output for each set of sampled evolutionary parameters during the MCMC chain. We can use these migration histories to assess mixing over migration histories analogously to the migration histories output when running an exact structured coalescent sampler using migration history summary statistics. S5 Fig shows trace plots of the migration frequency and deme branch lengths sampled using MASCOT. These trace plots are very similar to the corresponding trace plots using StructCoalescent (S2 Fig) with migration counts exploring similar ranges of values and stable branch lengths assigned to each deme. Using MASCOT, a median of 13 migration events (95% CI [10, 21]) were observed in a migration history, similar to the median of 13 migration events (95% CI [9, 18]) observed using StructCoalescent. Slight differences in the credible intervals of each method may be related to the approximation used by MASCOT, or the method used to estimate migration event locations and types.

Finally, we compare the inferences for evolutionary parameters drawn using MASCOT to those from our initial StructCoalescent analysis. S6 Fig shows empirical density estimates of the marginal posterior distribution of each evolutionary parameter, inverting effective population size samples from MASCOT to obtain coalescent rate estimates. Given that MASCOT is an approximation to the structured coalescent, the target posterior distributions of StructCoalescent and MASCOT are slightly different, although the posterior distributions are similar. The mode of each marginal empirical posterior distribution falls at the same sampled value although there are some visual differences between the tail behaviours for the two methods StructCoalescent tends to place slightly greater posterior mass at the mode of the distribution, with consequently lighter tails than MASCOT. These differences in tail behaviour may either be caused by StructCoalescent having fewer samples from regions of very low posterior density, or differences in the posterior from the MASCOT approximation.

Overall, the inferences drawn for this dataset using both StructCoalescent and MASCOT are extremely close to each other. This provides reasonable validation that our StructCoalescent methodology is successfully drawing samples from the desired posterior distribution and increases our confidence that our empirically-derived prior distributions are reasonable for inference under the structured coalescent.

### Determining the phylogenetic origin of Avian Influenza in Mexico

Avian influenza (AIV) is a pathogen common to a range of bird species which has been identified globally, with intercontinental spread likely to be caused by long-range migrations of wild birds [70]. The transmission between species and across locations in Central and North America in the early 2000s was previously described based on 133 AIV genomes [71]. We focus on the study of transmission between bird orders, with the 133 genomes distributed across four bird orders: Anseriformes (ANS), Charardiiformes (CHA), Galliformes (GAL) and Passeriformes (PAS), as well as transmission to domestic poultry in a more recent Mexican Outbreak (MEX). A dated phylogeny was inferred using a maximum likelihood method and geographic and interspecies spread was modelled as a discrete character using a DTA model [71]. There have been at least two previous reanalyses of this data using BASTA to assess interspecies spread between orders of birds [30], and a range of approximations to the structured coalescent to assess the geographic spread [32].

We reanalyse this previously-published data under the exact structured coalescent taking a dated maximum clade credibility tree as input [32] (S7 Fig). Unlike other analyses presented in this work, we associate each AIV sample with the order of bird from which it was sampled rather than its geographic location. Migration events are now interpreted as an interspecies transmission event rather than a geographic migration, but the structured coalescent remains a reasonable model for this spread. We completed two analyses of this dataset with the first analysis consisting of five MCMC chains using our default prior distributions, $\theta_{i}∼\text{Gamma}(1,0.99)\text{ and }\lambda_{ij}∼\text{Gamma}(1,52.14).$ The second analysis also consisted of five MCMC chains but instead using exponentially-distributed priors with rate 1 to match a previous analysis using BASTA [30]. Both analyses place almost identical priors on coalescent rates, although the migration rate priors differ substantially between the analyses. We used the same initial migration histories for both analyses with migration histories sampled from the DTA probability density (Equation 4) and initial evolutionary parameters set to the prior mean of our default priors (Equation 9). The initial backwards-in-time migration rate values slightly favour our first analysis using our default Gamma priors, but an Exp(1) distribution still observes high probability density at these values. Each MCMC chain was terminated at 48 hours of run time, with joint samples of evolutionary parameters saved every 100 iterations and migration histories saved every 1000 iterations.

**Table 2:** ESS results for evolutionary parameter estimates in the analysis of the AIV phylogeny using both the default prior distributions and Exp(1) prior distributions.

	Default priors					Exp(1) priors				
	Run 1	Run 2	Run 3	Run 4	Run 5	Run 1	Run 2	Run 3	Run 4	Run 5
**Completed iterations**										
*N*	2,931,100	2,965,400	2,925,900	2,965,900	2,951,600	6,870,000	5,909,200	5,408,500	5,308,800	6,171,300
**Minimum ESS**										
$\theta_{\text{min}}$	928	1122	790	1039	1001	43	90	41	60	110
$\lambda_{\text{min}}$	287	655	565	507	266	34	41	28	36	50
**Joint ESS**										
*Θ*	1095	1342	1215	1309	1183	98	258	108	170	108
*Λ*	1208	1353	1277	1247	1232	130	179	201	164	137
( *Θ* , *Λ* )	1243	1446	1354	1320	1284	184	237	263	212	177

The MCMC chains using our default priors completed a mean of 2,947,980 iterations, approximately half as many as the MCMC chains using Exp(1) priors which had a mean of 5,933,560 iterations (Table 2). Despite this large discrepancy, we find that MCMC chains using our default priors contain substantially lower autocorrelation with much higher ESS. Almost every MCMC sample drawn using Exp(1) priors identified at least one parameter with ESS below 100 and joint ESS values below 300. We also identified clear signs for a lack of convergence in the Exp(1) samples with a multivariate $\hat{R}$ of 4.48 and 21 out of 25 evolutionary parameters with univariate $\hat{R}$ exceeding 1.1. In contrast, our default prior MCMC chains observed just two parameters with ESS dropping below 300, and every other evolutionary parameter returning an ESS exceeding 500 in every run. Joint ESS was similarly much higher with every run exceeding a joint ESS of 1,000 and there were no indications of a lack of convergence with a multivariate $\hat{R}$ value of 1.025 and all univariate $\hat{R}$ values below 1.02. Full results for ESS and $\hat{R}$ values are available in S8 and S9 Tables. We also see a lack of convergence over migration histories for the Exp(1) prior MCMC chains. Trace plots of the total count of migration events in each migration history (A Fig) show high autocorrelation between migration histories with clear trends in the traces. We also fail to explore consistent ranges of migration counts in each run, with runs 1 and 5 typically exploring migration counts between 200 and 400, whilst runs 2, 3 and 4 typically remain between 100 and 200 migration events. There are similar issues with high autocorrelation and inconsistent regions of exploration in the stacked trace plots of branch lengths in each deme (B Fig). Together, these indicate that migration histories are mixing slowly and running these MCMC chains until convergence would require significantly longer runs with many more days of computation time.

Even accounting for the failure to converge, we believe that these results are sufficient evidence that Exp(1) priors are not a suitable choice for this dataset with an unrestricted migration model. Exp(1) priors may be reasonable if some migration paths are restricted, either a priori or during the run using, for example Bayesian stochastic search variable selection [28], although we do not test this here. The MCMC chains have repeatedly explored migration histories containing many more migration events than we would expect - a parsimonious reconstruction requires just 8 migration events compared with a mean of 214 sampled migration events over the five MCMC runs. We believe that this discrepancy is sufficiently large that, even if we were to run these MCMC chains for many more iterations until convergence was achieved, we would not recover biologically-reasonable results. The high number of inferred migration events is likely induced by the migration rates prior. Given that *d* = 5 is the number of demes and *L* ≈ 104 is the total branch length in years, the Exp(1) prior implies a prior expected number of migration events under DTA of  ( *d* − 1 ) *L* ≈ 417. Whilst this will not be the same as the expected number of migration events under the structured coalescent, the two should be positively correlated and we can see that Exp(1) priors support much higher numbers of migration events on this phylogeny. The large proportion of the migration history assigned to the Mexico Outbreak deme when using the Exp(1) prior is also unlikely to be correct (B Fig). Most sampled migration histories assign at least 20% of the history to this deme, with some samples extending to as much as 60% of the migration history. The Mexico H7N3 outbreak was first noted in June 2012 [71], and is very unlikely to have been present and unobserved in Mexico for a long period of time prior to this date.

**Fig 7:**
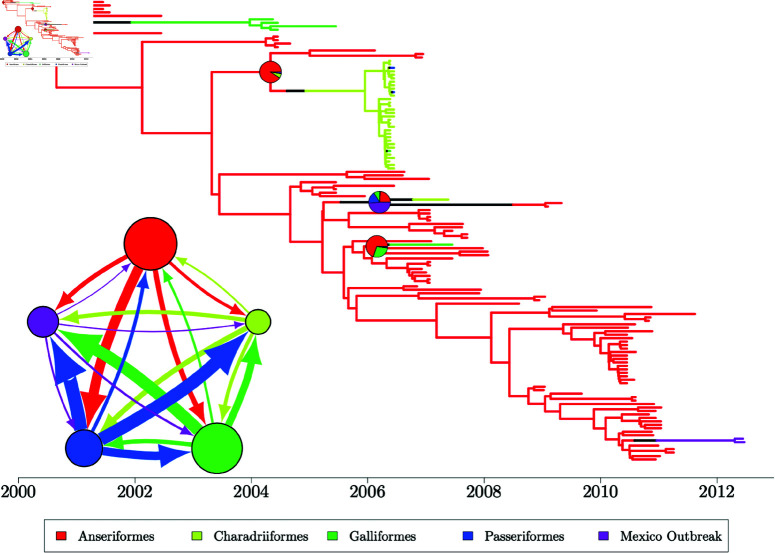
60% consensus migration history computed on all posterior migration history samples from the AIV analysis with default priors. Inset: migration model with 5 demes. The radius of each deme circle is proportional to the median inferred effective population size (inverse coalescent rate) for that deme and the width of an arrow connecting two demes is proportional to the magnitude of the backwards-in-time migration rate between the pair of demes.

Returning to the results from our default priors, we find little evidence to indicate a lack of convergence over migration histories. We infer a consistent number of migration events with most migration histories containing between 8 and 20 migration events, and have very consistent proportions of the migration history in each deme throughout all five runs (S9 Fig). There is no longer a large proportion of the migration history which falls into the purple Mexico outbreak deme, with almost all migration histories containing at least 80% of the history within the red Anseriformes deme. This may be an artefact caused by biased sampling of leaves, with 93 of the 133 leaves sampled from the Anseriformes deme, but is consistent with both a parsimonious reconstruction and the original results obtained using DTA [71].

In the analysis using BASTA with Exp(1) priors [30], the deme at the root was very uncertain with almost uniform probability that the root may fall into any deme. In the same paper, a DTA model was applied to the same data, which recovered probability 1 of the root falling into the red Anseriformes deme. We differ from both of these results, with high probability that the root falls into the Anseriformes deme, but maintaining a smaller posterior probability of falling into any of the other four demes (Fig 7). In line with the deme branch length plots (S9 Fig), we find the majority of the migration history to be coloured in red with little variability at internal coalescent events, but observe some uncertainty in the precise position of migration events along branches and the deme at some coalescent events.

### The seventh pandemic of cholera

The bacteria *Vibrio cholerae* is the causative agent of cholera, an infectious disease often thought of as only being historically important despite the fact that the seventh pandemic officially started in 1961 and is still ongoing [72]. Several waves of global transmission in the seventh cholera pandemic were described on the basis of a comparison between 154 whole genomes of *V. cholerae* [73]. A later analysis further clarified the global migration history of the seventh cholera pandemic in particular by adding many genomes from China [74]. This study was based on a total of 260 genomes sampled between 1957 and 2010 from 11 locations: Africa (AFR), Bahrain (BHR), Europe (EUR), Haiti (HTI), Indonesia (IDN), South America (SA), South Asia (SAS), Southeast Asia (SEA), China (CHN), Pakistan (PAK) and Nepal (NPL). A maximum parsimony approach [48] was used to reconstruct a migration history, resulting in a minimum of 37 migration events, of which 18 were between an unambiguous pair of demes [74]. The greatest number of migrations were found to have occurred from South Asia into China, and vice versa.

We reanalysed this previously-published data using the structured coalescent to add explicit sampling of migration histories to the original analysis. We take as input the same dated phylogeny (S10 Fig) and ran eleven MCMC chains with initial migration histories sampled from the DTA probability density such that each run uses each unique deme at the root. Our preliminary analysis using our default priors (Equation 9) failed to fully converge with some sharp changes in migration count throughout the MCMC runs (S11 Fig). The migration histories also frequently featured high prevalence of the Haiti deme with many migration histories having at least 10% of the history occurring in Haiti (S12 Fig). This requires many migration events into and out of Haiti as well as contradicting the scientific consensus that the seventh pandemic cholera entered Haiti in early 2010 following at least a century without infection [75,76]. In light of these issues, we repeated our analysis using a more conservative prior on migration rates to reduce the prior expected number of migration events. We found that this resulted in more parsimonious migration histories and we were able to identify some known events in the spread of seventh pandemic cholera.

The rate of our default prior on migration rates (Equation 9) is specified using the number of demes *d*, total branch length of the phylogeny *L* and a prior belief about the number of migration events *M*. By default, we set *M* to be the number of required migration events in a maximum parsimony migration history, in this case *M* = 37 migration events. We approximately halved *M* to a value of *M* = 20, resulting in reduced prior distributions $\theta_{i}∼\text{Gamma}(1,5.59)\text{ and }\lambda_{ij}∼\text{Gamma}(1,632.67).$ Each of the eleven MCMC runs completed around 2,500,000 iterations and obtained reasonably high quality samples. Every evolutionary parameter sample contained reasonably low autocorrelation with ESS values of at least 250 and mean per-parameter ESS of 1,921. Joint ESS values were similarly high with a minimum joint ESS of 2,901. All samples also converged well to a consistent distribution with $\hat{R}$ statistics of at most 1.03. Full results for ESS and $\hat{R}$ are available in S10 and S11 Tables. We also find no evidence to indicate poor convergence over migration histories. Sampled migration histories tend to feature between 45 and 50 migration events with occasional drops to 37 migration events, matching with the number of migration histories required for a maximum parsimony migration history (A Fig). We also have extremely consistent proportions of the migration history assigned to each deme, and notably observe very small proportions of the Haitian deme unlike our preliminary analysis (B Fig).

**Fig 8:**
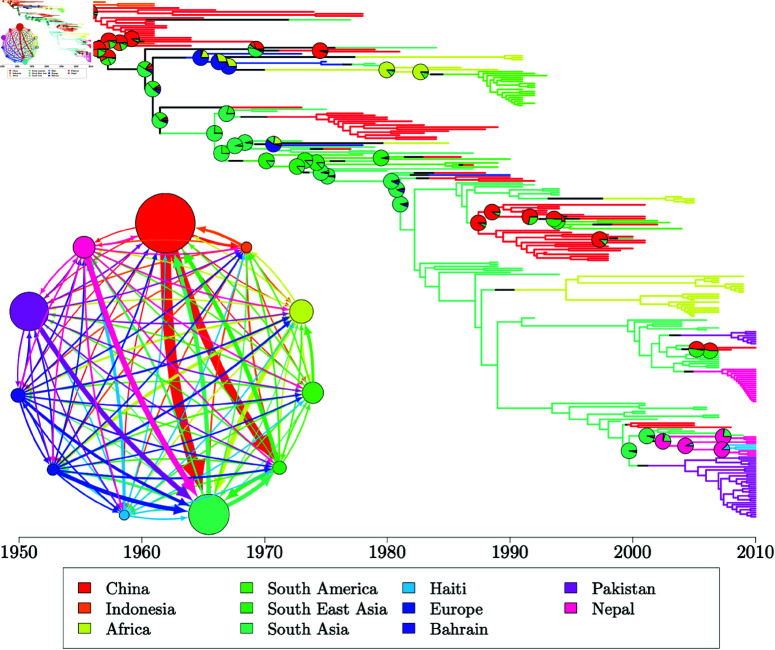
60% consensus migration history based on all migration histories sampled across the eleven MCMC chains in our cholera analysis. Inset: migration model with 11 demes. The radius of each deme circle is proportional to the median inferred effective population size for that deme and the width of an arrow connecting two demes is proportional to the magnitude of the backwards-in-time migration rate between the pair of demes.

Fig 8 shows a 60% consensus migration history with the median inferred effective population sizes and backwards-in-time migration rates shown inset. We find very high probability of the root arising in Asia with 92% of the sampled migration histories identifying the root in Indonesia, 4.8% in South East Asia, 1.8% in China and the remaining 1.4% split between the remaining demes. There remains some uncertainty about the precise origin within Asia, but the inference is likely to depend on the sampling scheme for lineages in the phylogeny. Most of the lineages which are isolated close to the root are obtained either from China or Indonesia, and the resulting migration history would require a strong migration process to migrate away from either deme in such a short period of time.

We now focus on two well-documented transmission events which are recovered by our analysis: the introduction of seventh pandemic cholera into Haiti, and the introduction of seventh pandemic cholera into South America. Cholera is widely believed to have been reintroduced into Haiti after an earthquake in early 2010 by Nepalese aid workers following at least a century since the previous Haitian cholera outbreak [75,76]. We infer a single transmission event into Haiti between 2007 and 2008 (A Fig), between 2 and 3 years earlier than would be expected.

Seventh pandemic cholera was identified in South America in January 1991 [77,78], although the origin of the transmission was initially unclear. It was originally suggested that cholera was reintroduced into South America from Asia by international trade ships, but more recent work has found genetic similarities between the African and South American strains which are not present in Asian samples [79]. We infer a transmission of cholera from Africa into South America between 1983 and 1986 (B Fig), between 5 and 8 years earlier than the true transmission event although we accurately infer the likely source of transmission. In both cases, we have estimated the transmission times earlier than the literature suggests, likely caused by an explosive population growth of cholera following introduction. There was no endemic cholera population in either Haiti or South America prior to reintroduction and consequently we would expect little immunity and rapid growth. This violates the assumption of fixed deme sizes (equivalently fixed coalescent rates) in the structured coalescent and makes precise inference in these regions of the phylogeny unreliable.

We identify China as the largest population of seventh pandemic cholera with other large populations in South Asia and Pakistan (Fig 8, inset). We also find the greatest backwards-in-time migration rates to be those from China into South Asia and South East Asia, with most of the largest backwards-in-time migration rates targeting South East Asia. We have less capacity to distinguish between the smallest inferred migration rates and instead consider the number of migration events backwards-in-time (S15 Fig). The number of migration events between a pair of deme is usually positively correlated with the migration rate with higher migration rates usually inducing larger quantities of migration events. If many samples are drawn from the same deme, we may still observe many migration events even with low migration rates, especially if the samples are separated into multiple sampling groups. In line with the findings of the original analysis [74], we identify the greatest number of migration events between China, South Asia and Southeast Asia with relatively few migration events between other pairs of demes.

## Discussion

We have presented a new divide and conquer framework [36,37] for Bayesian inference under the exact structured coalescent in which the inference of a dated phylogenetic tree is separated from the phylogeographic inference of migration histories conditional on this phylogeny. Our modular approach differs from existing approaches which either approximate the structured coalescent, or use MCMC sampling to explore the full space of dated structured phylogenetic trees in one step. We also present a MCMC sampling scheme capable of exploring the joint space of migration histories and evolutionary parameters conditional on a fixed phylogeny by generating local updates to the migration history using discrete trait analysis (DTA).

We illustrated the use of our inferential framework by application to a range of simulated and previously-published empirical datasets. Simulations indicate reasonable performance for our method for phylogenetic trees with 1000 leaves and up to 10 demes. Most runs converged within 48 hours, producing accurate estimates of evolutionary parameters for all but the smallest migration rates. Applications to previously-published *S. aureus*, avian influenza and cholera datasets allowed us to replicate some previously-described conclusions about the spread of these pathogens [67,71,74]. Bayesian inference using the structured coalescent is highly prior dependent, typically with high correlation between the number of inferred migration events and the expectation of migration rate priors. The avian influenza analysis illustrates this dependence with a comparison between our default prior distributions and a previously-described analysis using less conservative exponential priors [30]. The less conservative priors showed clear signs of a lack of convergence as well as supporting many more migration events than would be expected.

Separating the inference of a dated phylogeny from the phylogeographic inference reduces the size of the target space within each module, making MCMC target spaces much smaller and easier to explore. In particular, this aids us in scaling to higher numbers of demes and phylogenies containing more leaves than existing methods. However, separating the inference into modules introduces new challenges to perform rigorous analyses. Most existing methods to infer dated phylogenetic trees from genomic data are designed for inference with unstructured populations [80], using assumptions incompatible with the structured coalescent. This can introduce misspecification into our inference where the phylogeny is inferred under a different model to that assumed in our phylogeographic inference, introducing biases in inferences. For example, when simulating dated structured phylogenetic trees using the structured coalescent, separating lineages into multiple subpopulations reduces the total coalescent rate of a sample, consequently changing the distribution of branch lengths. It is less clear how population structure may affect the inference of a dated phylogeny from genomic data where mutation rates may be independent of the location of a lineage.

Furthermore, we have presented results conditioning on a single inferred phylogeny which we treat as the ground truth. Ignoring phylogenetic uncertainty in this way will inherently reduce the uncertainty in evolutionary parameter estimates compared with an integrated approach to phylogeography in which the dated phylogeny is simultaneously inferred. One approach to incorporate phylogenetic uncertainty into StructCoalescent is to perform independent analyses on a sampled of dated phylogenies [81] and combine inferences across MCMC runs using an importance sampling approach [82]. However, in a typical phylogeographic study we would expect the genomic data to be quite strongly informative about the ancestral relationships between genomes. Assuming a single dated tree should therefore be less of an issue than for studies on smaller evolutionary scales, such as analysis of densely sampled outbreaks [83].

There are several possible extensions to this work. Our MCMC framework uses an approximation to the structured coalescent to update the the migration history locally. Whilst we currently present this framework with a fixed dated phylogeny, the phylogeny could also be updated by including standard MCMC operators, such as those used by MultiTypeTree. DTA is a reasonably tractable model from which we are able to sample explicit migration histories, but replacing DTA with a more sophisticated approximation to the structured coalescent could result in migration history proposals which more closely resemble a direct sample from the structured coalescent. In this case, typical acceptance probabilities for a given subtree should increase, in turn permitting larger subtrees to be considered and potentially improving the rate of mixing. Most existing approximations such as BASTA [30] and MASCOT [31] integrate out the migration history and sample demes at fixed points, usually coalescent events. Such approximations could be applied locally to sample deme configurations within a subtree although Poisson bridges would still be required to obtain explicit migration event types and positions. All of the simulations presented in this work use relatively low, balanced migration rates between demes as well as even rates of acquiring samples from each deme. Further work could test the robustness of our approach when these situations are not satisfied, for example in the presence of higher migration rates, or strong source/sink dynamics with many samples obtained from the source deme.

DTA, BASTA and MASCOT each include implementations of Bayesian stochastic search variable selection (BSSVS) which can be used to reduce the number of migration rates which are inferred during an MCMC run [45]. BSSVS could be added to our inferential framework via a ‘coin-flip’ operator in which a single migration rate is selected at random to change state between either being switched off and set to rate 0, or switched on and being resampled away from 0. Implementing BSSVS in this way does not fundamentally change the Gibbs moves in use for evolutionary parameter updates however there may be restrictions where a rate can not be switched off if there are any observed migration events of that type. BASTA and MASCOT both avoid this problem by integrating over migration histories and hence always being able to freely flip any migration rate which does not break the irreducibility of the migration model.

The structured coalescent can be viewed as a time-homogeneous compartmental model with merger (or splitting) events restricted to occur entirely with a single subpopulation. More general compartmental models can relax this assumption, allowing merger events between lineages in different demes [84,85]. Bayesian inference for compartmental models with piecewise-continuous migration processes could be reasonable candidates for local updates to the migration history with Poisson bridges used to sample trajectories along branches of a phylogeny.

## Supporting Information

S1 Text Prior conjugacy. (PDF)

S1 Table Effective sample sizes estimates for evolutionary parameters for an application to a single simulated structured phylogeny. (PDF)

S2 TableGelman–Rubin $\hat{R}$ statistics for evolutionary parameters for an application to a single simulated structured phylogeny.The first column gives the $\hat{R}$ value for the coalescent rate in each deme whilst the remaining columns give the $\hat{R}$ values for backwards-in-time migration rates between pairs of demes. The row gives the source deme for a migration rate and the column gives the target deme (backwards-in-time). The greatest $\hat{R}$ values are highlighted in **bold**.(PDF)

S3 TableSummary of convergence diagnostics for evolutionary parameters for an application to multiple simulated structured phylogenies.Convergence diagnostics are separated by number of demes and degree of heterochronicity in leaf sampling, and reported completed iterations and joint ESS values are means taken over all pairs of MCMC chains of that type which converge ($\hat{R}\leq 1.2$).(PDF)

S1 Fig Dated phylogeny used as input for the MRSA analysis. (PDF)

S4 Table Effective sample size estimates for evolutionary parameters for the MRSA analysis. (PDF)

S5 TableGelman–Rubin $\hat{R}$ statistics for evolutionary parameters for the MRSA analysis.The first column gives the $\hat{R}$ value for the coalescent rate in each deme whilst the remaining columns give the $\hat{R}$ values for backwards-in-time migration rates between pairs of demes. The row gives the source deme for a migration rate and the column gives the target deme (backwards-in-time). The greatest $\hat{R}$ values are highlighted in **bold**.(PDF)

S2 Fig Migration history summary statistics for the MRSA analysis. (**a**) Trace plot of the total migration count. The black dashed line indicates the number of required migration events for a maximum parsimony migration history. (**b**) Stacked trace plot of the proportion of the migration history falling into each deme.
(PDF)

S3 Fig Kernel density estimates of the posterior density of backwards-in-time migration rates, separated by origin deme. The black line indicates the prior density for all migration events.
(PDF)

S4 Fig Trace plots for MultiTypeTree MCMC runs. (**a**) Log-posterior density evaluations with gamma-distributed priors matching our default priors. (**b**) Posterior migration counts with gamma-distributed priors matching our default priors. (**c**) Log-posterior density evaluations with lognormal priors matching the MultiTypeTree default priors. (**d**) Posterior migration counts with lognormal priors matching the MultiTypeTree default priors.

S6 Table ESS for the MASCOT analysis of the MRSA dataset. (PDF)

S7 TableGelman–Rubin $\hat{R}$ statistics for the MASCOT analysis of the MRSA dataset.Greatest $\hat{R}$ values are highlighted in **bold**.(PDF)

S5 Fig Trace plots of migration history summary statistics for the MASCOT analysis of the MRSA dataset. (**a**) Trace plot of the total migration count. The black dashed line indicates the number of required migration events for a maximum parsimony migration history. (**b**) Stacked trace plot of the proportion of the migration history falling into each deme across the five MCMC samples.
(PDF)

S6 Fig Empirical posterior density estimates for evolutionary parameters sampled using Local DTA analysis (shown in red) and MASCOT (shown in blue). (PDF)

S7 Fig Dated phylogeny used as input for the AIV analysis. (PDF)

S8 Table Effective sample size estimates for evolutionary for the AIV analysis with default gamma-distributed priors (left) and Exp(1) priors (right). (PDF)

S9 TableGelman–Rubin $\hat{R}$ statistics for evolutionary parameters for the AIV analysis with (a) default priors; (b) Exp(1) priors.The first column gives the $\hat{R}$ value for the coalescent rate in each deme whilst the remaining columns give the $\hat{R}$ values for backwards-in-time migration rates between pairs of demes. The row gives the source deme for a migration rate and the column gives the target deme (backwards-in-time). The greatest $\hat{R}$ values are highlighted in **bold**.(PDF)

S8 Fig Migration history summary statistics for the AIV analysis with Exp(1) priors. (**a**) Trace plot of the total migration count. The black dashed line indicates the number of required migration events (*M* = 8) for a maximum parsimony migration history. (**b**) Stacked trace plot of the proportion of the migration history falling into each deme.
(PDF)

S9 Fig Migration history summary statistics for the AIV analysis with default priors. (**a**) Trace plot of the total migration count. The black dashed line indicates the number of required migration events (*M* = 8) for a maximum parsimony migration history. (**b**) Stacked trace plot of the proportion of the migration history falling into each deme.
(PDF)

S10 Fig Dated phylogeny used as input for the cholera analysis. (PDF)

S11 Fig Migration history summary statistics for the Cholera analysis with default priors. (**a**) Trace plot of the total migration count. The black dashed line indicates the number of required migration events (*M* = 37) for a maximum parsimony migration history. (**b**) Stacked trace plot of the proportion of the migration history falling into each deme.
(PDF)

S12 Fig 60% consensus migration history for the preliminary Cholera analysis using default priors. (PDF)

S10 Table Effective sample size estimates for evolutionary parameters for the cholera analysis.

S11 TableGelman–Rubin $\hat{R}$ statistics for evolutionary parameters for the cholera analysis.The first column gives the $\hat{R}$ value for the coalescent rate in each deme whilst the remaining columns give the $\hat{R}$ values for backwards-in-time migration rates between pairs of demes. The row gives the source deme for a migration rate and the column gives the target deme (backwards-in-time).(PDF)

S13 Fig Migration history summary statistics for the cholera analysis with reduced priors. (**a**) Trace plot of the total migration count. The black dashed line indicates the number of required migration events (*M* = 37) for a maximum parsimony migration history. (**b**) Stacked trace plot of the proportion of the migration history falling into each deme.
(PDF)

S14 Fig Sections of the 60% consensus migration history for the cholera analysis focussed on transmissions into (a) Haiti; (b) South America. (PDF)

S15 Fig Median number of backwards-in-time migration events between pairs of demes in the cholera analysis. The width of each arrow denotes the relative frequency with which that migration type was observed.
(PDF)
