# Supplementary material for: Bayesian Inference of Pathogen Phylogeography using the Structured Coalescent Model
Source: PLoS Comput Biol. 2025 Apr 21;21(4):e1012995. doi: 10.1371/journal.pcbi.1012995 (PMC12040344; doi:10.1371/journal.pcbi.1012995)
Supplement: S1 Text — (PDF) [file pcbi.1012995.s001.pdf]

Supplementary Text S1: Prior Conjugacy

We illustrate the conjugacy of Gamma-distributed priors for both coalescent rates and backwards-in-time migration rates with the structured coalescent likelihood by deriving the posterior parameters for the Gibbs updates (Equation 10). Consider placing a collection of independent Gamma-distributed priors on evolutionary parameters,

$$\theta_i \sim \text{Gamma}(\alpha_i, \beta_i), \qquad \lambda_{ij} \sim \text{Gamma}(\alpha_{ij}, \beta_{ij}), \tag{S1}$$

with arbitrary prior parameters $\{\alpha_i, \beta_i\}_{i=1}^{d}$ and $\{\alpha_{ij}, \beta_{ij}\}_{\substack{i,j=1 \\ j \neq i}}^{d}$. The probability density of coalescent rate θ_i conditional on all other evolutionary parameters and the structured genealogy is then given by

$$\begin{aligned} p(\theta_i | \mathbf{\Theta}^{-(i)}, \mathbf{\Lambda}, \mathcal{H}, \mathcal{T}) &= \frac{p(\mathcal{H}, \mathcal{T} | \theta_i, \mathbf{\Theta}^{-(i)}, \mathbf{\Lambda}) p(\theta_i, \mathbf{\Theta}^{-(i)}, \mathbf{\Lambda})}{p(\mathbf{\Theta}^{-(i)}, \mathbf{\Lambda}, \mathcal{H}, \mathcal{T})} \\ &= p(\theta_i) p(\mathcal{H}, \mathcal{T} | \mathbf{\Theta}, \mathbf{\Lambda}) \cdot \frac{p(\mathbf{\Theta}^{(-i)}, \mathbf{\Lambda})}{p(\mathbf{\Theta}^{-(i)}, \mathbf{\Lambda}, \mathcal{H}, \mathcal{T})} \\ &\propto p(\theta_i) P_{\text{SC}}(\mathcal{T}, \mathcal{H} | \mathbf{\Theta}, \mathbf{\Lambda}) \\ &\propto \theta_i^{\alpha_i + c_i - 1} \exp\left\{ -\theta_i \left(\beta_i + \sum_r \binom{k_{ir}}{2} \tau_r \right) \right\}, \end{aligned} \tag{S2}$$

which we identify as a gamma distribution

$$\theta_i | (\mathbf{\Theta}^{-(i)}, \mathbf{\Lambda}, \mathcal{H}, \mathcal{T}) \sim \Gamma\left(\alpha_i + c_i, \beta_i + \sum_r \binom{k_{ir}}{2} \tau_r \right). \tag{S3}$$

A similar argument can be applied to the probability density of each migration rate λ_{ij} conditional on all other evolutionary parameters and the structured genealogy,

$$\begin{aligned} p(\lambda_{ij} | \mathbf{\Theta}, \mathbf{\Lambda}^{-(i,j)}, \mathcal{H}, \mathcal{T}) &= \frac{p(\mathcal{H}, \mathcal{T} | \mathbf{\Theta}, \lambda_{ij}, \mathbf{\Lambda}^{-(i,j)}) p(\mathbf{\Theta}, \lambda_{ij}, \mathbf{\Lambda}^{-(i,j)})}{p(\mathbf{\Theta}, \mathbf{\Lambda}^{-(i,j)}, \mathcal{H}, \mathcal{T})} \\ &= p(\lambda_{ij}) p(\mathcal{H}, \mathcal{T} | \mathbf{\Theta}, \mathbf{\Lambda}) \cdot \frac{p(\mathbf{\Theta}, \lambda_{ij}, \mathbf{\Lambda}^{-(i,j)})}{p(\mathbf{\Theta}, \mathbf{\Lambda}^{-(i,j)}, \mathcal{H}, \mathcal{T})} \\ &\propto p(\lambda_{ij}) P_{\text{SC}}(\mathcal{T}, \mathcal{H} | \mathbf{\Theta}, \mathbf{\Lambda}) \\ &\propto \lambda_{ij}^{\alpha_{ij} + m_{ij} - 1} \exp\left\{ -\lambda_{ij} \big(\beta_{ij} + \sum_r k_{ir} \tau_r \big) \right\}, \end{aligned} \tag{S4}$$

which we again identify as a Gamma distribution

$$\lambda_{ij} | (\mathbf{\Theta}, \mathbf{\Lambda}^{-(i,j)}, \mathcal{H}, \mathcal{T}) \sim \Gamma\big(\alpha_{ij} + m_{ij}, \beta_{ij} + \sum_r k_{ir} \tau_r \big). \tag{S5}$$
