# Supplementary material for: Bayesian Inference of Pathogen Phylogeography using the Structured Coalescent Model
Source: PLoS Comput Biol. 2025 Apr 21;21(4):e1012995. doi: 10.1371/journal.pcbi.1012995 (PMC12040344; doi:10.1371/journal.pcbi.1012995)
Supplement: S1 Table — (PDF) [file pcbi.1012995.s002.pdf]

	Run 1	Run 2	Run 3	Run 4	Run 5	Run 6	**Min**	**Max**	**Mean**
**Coalescent rates**									
θ_1	1110	979	871	896	934	992	**871**	**1110**	**964**
θ_2	1284	1119	1401	956	929	1306	**929**	**1401**	**1166**
θ_3	781	775	1047	974	1004	823	**775**	**1047**	**901**
θ_4	1156	1154	987	970	909	1036	**909**	**1156**	**1035**
θ_5	927	1270	755	778	962	1036	**755**	**1270**	**955**
θ_6	858	1025	991	1445	1185	1399	**858**	**1445**	**1150**
**Migration rates**									
$\lambda_{2,1}$	953	1024	1257	824	997	935	**824**	**1257**	**998**
$\lambda_{3,1}$	1004	841	927	907	1293	757	**757**	**1293**	**955**
$\lambda_{4,1}$	1153	849	759	795	1077	832	**759**	**1153**	**911**
$\lambda_{5,1}$	1234	1015	726	849	949	871	**726**	**1234**	**941**
$\lambda_{6,1}$	852	1003	830	964	1043	729	**729**	**1043**	**904**
$\lambda_{1,2}$	856	1084	967	810	963	1269	**810**	**1269**	**992**
$\lambda_{3,2}$	794	1158	704	728	1127	1033	**704**	**1158**	**924**
$\lambda_{4,2}$	1360	1170	865	1334	1184	1079	**865**	**1360**	**1165**
$\lambda_{5,2}$	918	686	832	879	1153	829	**686**	**1153**	**883**
$\lambda_{6,2}$	1123	897	837	755	1051	1217	**755**	**1217**	**980**
$\lambda_{1,3}$	807	971	835	1008	942	775	**775**	**1008**	**890**
$\lambda_{2,3}$	937	899	1013	1074	923	939	**899**	**1074**	**964**
$\lambda_{4,3}$	822	740	1024	850	1144	1172	**740**	**1172**	**959**
$\lambda_{5,3}$	957	789	783	1570	960	1365	**783**	**1570**	**1071**
$\lambda_{6,3}$	873	808	836	692	1254	824	**692**	**1254**	**881**
$\lambda_{1,4}$	549	763	844	773	746	659	**549**	**844**	**722**
$\lambda_{2,4}$	1067	773	870	893	1048	885	**773**	**1067**	**923**
$\lambda_{3,4}$	843	785	968	781	761	1015	**761**	**1015**	**859**
$\lambda_{5,4}$	745	841	915	687	998	851	**687**	**998**	**840**
$\lambda_{6,4}$	704	741	736	1002	885	763	**704**	**1002**	**805**
$\lambda_{1,5}$	832	1143	905	828	1148	808	**808**	**1148**	**944**
$\lambda_{2,5}$	850	795	721	790	999	826	**721**	**999**	**830**
$\lambda_{3,5}$	742	690	783	1159	1049	1084	**690**	**1159**	**918**
$\lambda_{4,5}$	853	842	1057	760	820	871	**760**	**1057**	**867**
$\lambda_{6,5}$	865	918	858	807	1208	811	**807**	**1208**	**911**
$\lambda_{1,6}$	737	682	816	923	932	820	**682**	**932**	**818**
$\lambda_{2,6}$	1045	1030	899	817	1039	1074	**817**	**1074**	**984**
$\lambda_{3,6}$	1113	965	1077	971	1098	1115	**965**	**1115**	**1056**
$\lambda_{4,6}$	985	922	822	934	999	1058	**822**	**1058**	**953**
$\lambda_{5,6}$	914	779	941	783	1003	842	**779**	**1003**	**877**

Table S1: Effective sample sizes estimates for evolutionary parameters for an application to a single simulated structured phylogeny.
