# Supplementary material for: Bayesian Inference of Pathogen Phylogeography using the Structured Coalescent Model
Source: PLoS Comput Biol. 2025 Apr 21;21(4):e1012995. doi: 10.1371/journal.pcbi.1012995 (PMC12040344; doi:10.1371/journal.pcbi.1012995)
Supplement: S2 Table — The first column gives the R^ value for the coalescent rate in each deme whilst the remaining columns give the R^ values for backwards-in-time migration rates between pairs of demes. The row gives the source deme for a migration rate and the column gives the target deme (backwards-in-time). The greatest R^ values are highlighted in bold. (PDF) [file pcbi.1012995.s003.pdf]

	θ_x	$\lambda_{x,1}$	$\lambda_{x,2}$	$\lambda_{x,3}$	$\lambda_{x,4}$	$\lambda_{x,5}$	$\lambda_{x,6}$
$x = 1$	1.00076	—	1.00020	1.00067	1.00076	1.00114	1.00064
$x = 2$	1.00071	1.00030	—	1.00094	1.00038	1.00091	1.00080
$x = 3$	**1.00096**	1.00074	1.00334	—	1.00032	1.00011	1.00091
$x = 4$	1.00050	1.00128	1.00036	1.00095	—	1.00099	1.00119
$x = 5$	1.00068	**1.00191**	1.00040	1.00086	1.00089	—	1.00120
$x = 6$	1.00062	1.00101	1.00111	1.00144	1.00197	1.00039	—

Table S2: Gelman–Rubin $\hat{R}$ statistics for evolutionary parameters for an application to a single simulated structured phylogeny. The first column gives the $\hat{R}$ value for the coalescent rate in each deme whilst the remaining columns give the $\hat{R}$ values for backwards-in-time migration rates between pairs of demes. The row gives the source deme for a migration rate and the column gives the target deme (backwards-in-time). The greatest $\hat{R}$ values are highlighted in **bold**.
