# Supplementary material for: Bayesian Inference of Pathogen Phylogeography using the Structured Coalescent Model
Source: PLoS Comput Biol. 2025 Apr 21;21(4):e1012995. doi: 10.1371/journal.pcbi.1012995 (PMC12040344; doi:10.1371/journal.pcbi.1012995)
Supplement: S3 Table — Convergence diagnostics are separated by number of demes and degree of heterochronicity in leaf sampling, and reported completed iterations and joint ESS values are means taken over all pairs of MCMC chains of that type which converge (R^≤1.2). (PDF) [file pcbi.1012995.s004.pdf]

	2 demes	3 demes	4 demes	5 demes	6 demes	7 demes	8 demes	9 demes	10 demes
**Completed iterations** ($\times 1000$)									
Homochronous	923	1,869	3,386	4,082	4,750	4,809	4,580	3,938	5,207
Mild Heterochronicity	925	2,537	3,015	2,789	4,742	2,872	3,611	4,403	4,930
Moderate Heterochronicity	1,517	3,190	3,689	2,841	3,337	2,849	3,405	3,001	3,327
Strong Heterochronicity	1,846	3,007	2,889	3,135	3,500	3,678	3,589	3,788	3,867
**Multivariate** $\hat{R} \leq 1.2$									
Homochronous	5/5	5/5	5/5	5/5	3/5	5/5	5/5	4/5	5/5
Mild Heterochronicity	5/5	5/5	5/5	4/5	5/5	2/5	2/5	3/5	3/5
Moderate Heterochronicity	5/5	3/5	5/5	5/5	4/5	3/5	5/5	1/5	1/5
Strong Heterochronicity	5/5	5/5	5/5	5/5	5/5	4/5	3/5	5/5	2/5
**Joint ESS**									
Homochronous	819	1,564	2,403	2,980	2,392	3,145	2,811	3,263	3,122
Mild Heterochronicity	791	2,020	2,323	2,187	2,437	2,616	2,103	2,061	2,165
Moderate Heterochronicity	1,257	1,786	2,092	1,813	2,100	2,211	1,736	1,436	1,677
Strong Heterochronicity	1,621	2,033	1,975	1,989	1,683	1,611	1,723	1,551	1,341
**Minimum ESS** > 200									
Homochronous	5/5	5/5	5/5	5/5	5/5	5/5	5/5	5/5	5/5
Mild Heterochronicity	5/5	5/5	5/5	5/5	5/5	5/5	5/5	5/5	4/5
Moderate Heterochronicity	5/5	5/5	5/5	5/5	5/5	4/5	5/5	4/5	4/5
Strong Heterochronicity	5/5	5/5	5/5	5/5	5/5	5/5	5/5	5/5	3/5

Table S3: Summary of convergence diagnostics for evolutionary parameters for an application to multiple simulated structured phylogenies. Convergence diagnostics are separated by number of demes and degree of heterochronicity in leaf sampling, and reported completed iterations and joint ESS values are means taken over all pairs of MCMC chains of that type which converge ($\hat{R} \leq 1.2$).
