# Supplementary figures and images for: Bayesian Inference of Pathogen Phylogeography using the Structured Coalescent Model
Source: PLoS Comput Biol. 2025 Apr 21;21(4):e1012995. doi: 10.1371/journal.pcbi.1012995 (PMC12040344; doi:10.1371/journal.pcbi.1012995)

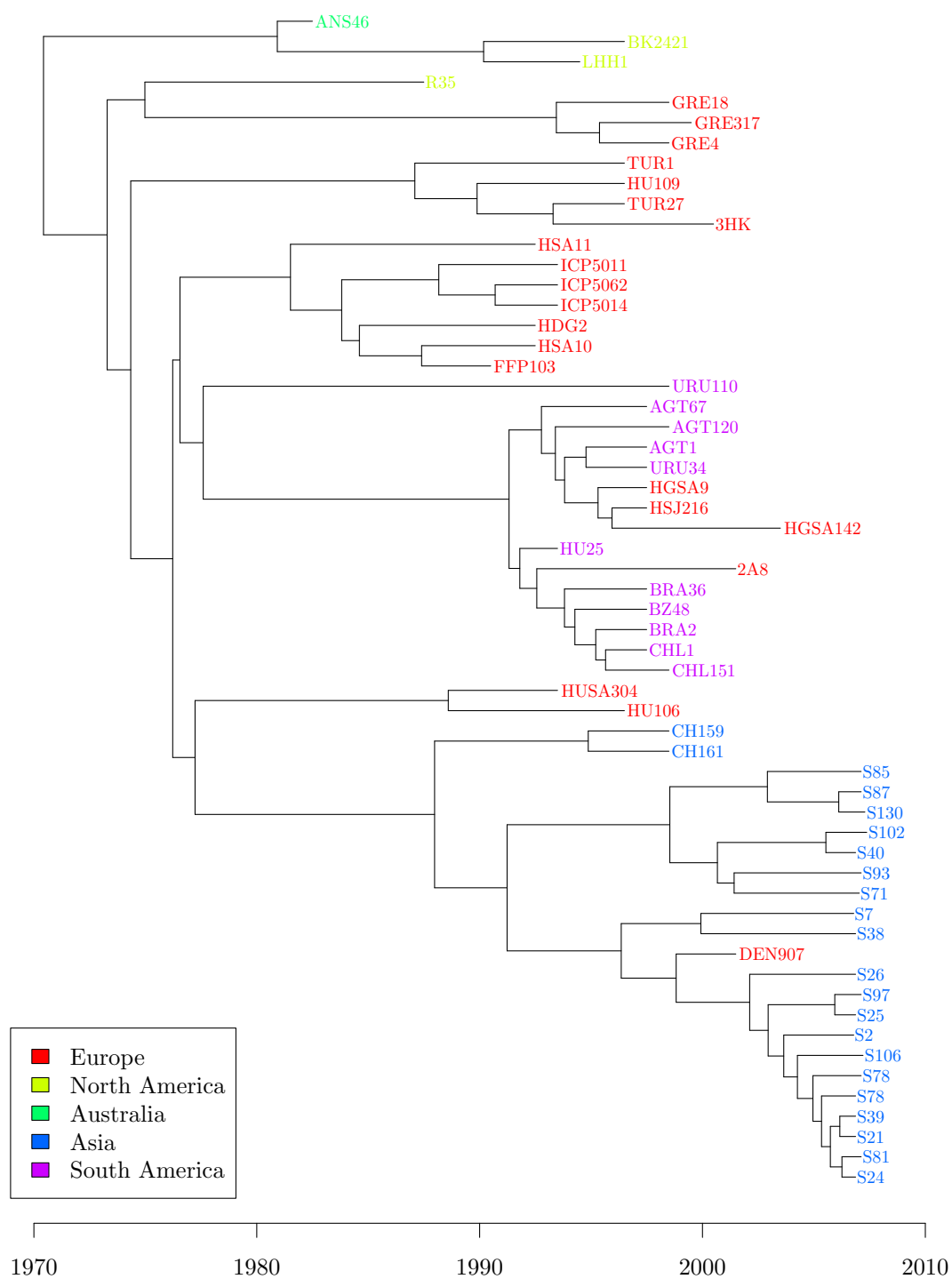

Figure S1: Dated phylogeny used as input for the MRSA analysis.

Supplement: S1 Fig — (PDF) [file pcbi.1012995.s005.pdf]

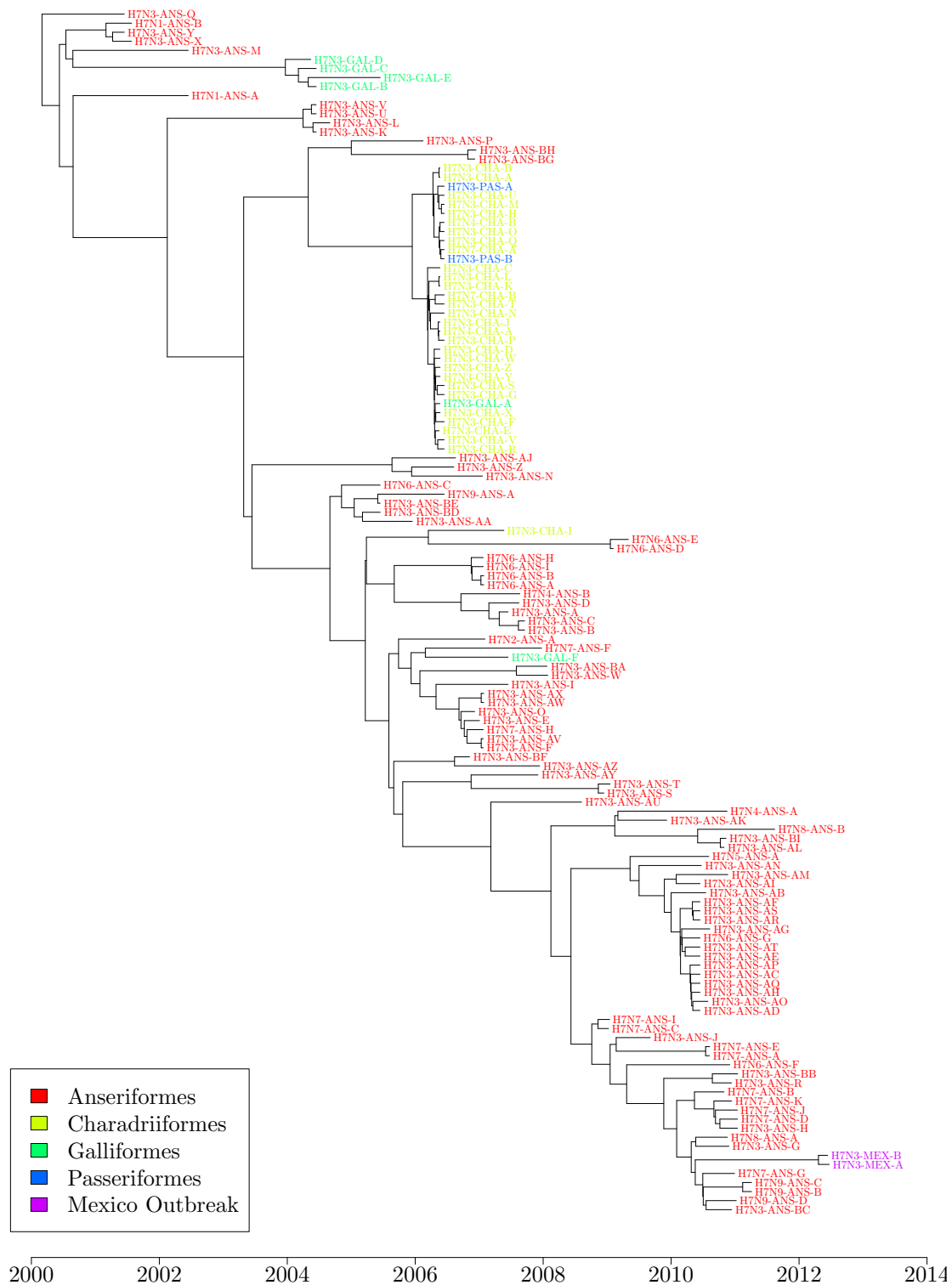

Figure S7: Dated phylogeny used as input for the AIV analysis.

Supplement: S7 Fig — (PDF) [file pcbi.1012995.s015.pdf]

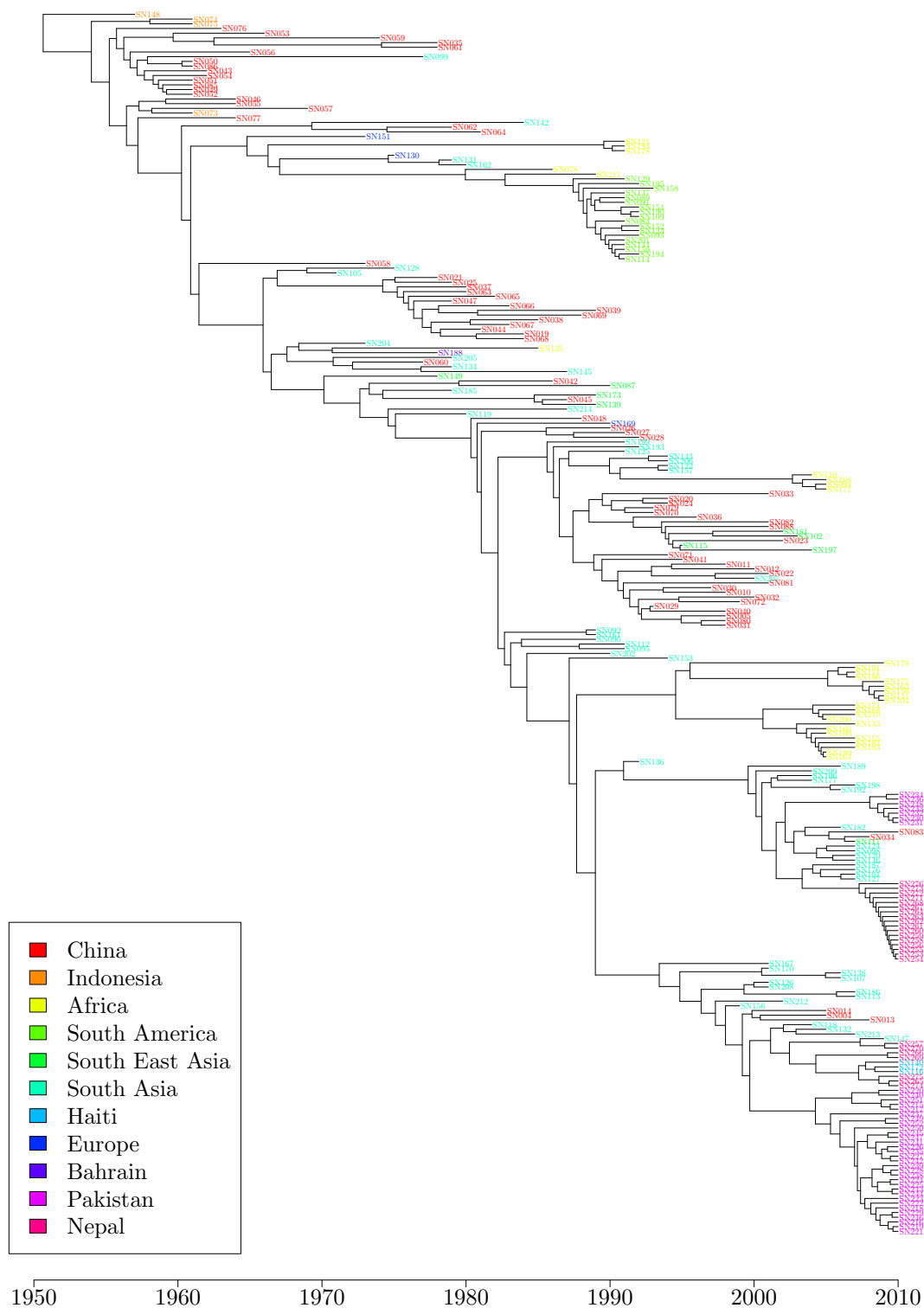

Figure S10: Dated phylogeny used as input for the cholera analysis.

Supplement: S10 Fig — (PDF) [file pcbi.1012995.s020.pdf]
