# Supplementary material for: Bayesian Inference of Pathogen Phylogeography using the Structured Coalescent Model
Source: PLoS Comput Biol. 2025 Apr 21;21(4):e1012995. doi: 10.1371/journal.pcbi.1012995 (PMC12040344; doi:10.1371/journal.pcbi.1012995)
Supplement: S4 Table — (PDF) [file pcbi.1012995.s006.pdf]

	Run 1	Run 2	Run 3	Run 4	Run 5
**Coalescent rates**					
θ_{EUR}	925	1237	1196	1033	866
θ_{NA}	1120	1149	1434	932	1128
θ_{AUS}	1057	917	968	816	1391
θ_{AS}	1436	1118	1560	1289	1277
θ_{SA}	1051	898	975	1128	1000
**Migration rates**					
$\lambda_{\text{NA,EUR}}$	1047	938	946	1034	933
$\lambda_{\text{AUS,EUR}}$	1696	1196	1173	1231	1066
$\lambda_{\text{AS,EUR}}$	988	964	908	1164	1075
$\lambda_{\text{SA,EUR}}$	1160	808	1034	858	883
$\lambda_{\text{EUR,NA}}$	807	779	1080	919	618
$\lambda_{\text{AUS,NA}}$	1249	1284	1252	1037	1018
$\lambda_{\text{AS,NA}}$	1324	890	1047	1049	875
$\lambda_{\text{SA,NA}}$	1046	1111	1210	932	1403
$\lambda_{\text{EUR,AUS}}$	378	748	474	537	315
$\lambda_{\text{NA,AUS}}$	1017	1173	1205	1193	1243
$\lambda_{\text{AS,AUS}}$	492	466	1708	1164	855
$\lambda_{\text{SA,AUS}}$	1071	1178	1033	854	1084
$\lambda_{\text{EUR,AS}}$	714	543	631	655	511
$\lambda_{\text{NA,AS}}$	1260	880	1325	1619	1027
$\lambda_{\text{AUS,AS}}$	1599	1279	1397	1068	1353
$\lambda_{\text{SA,AS}}$	1086	834	977	964	761
$\lambda_{\text{EUR,SA}}$	632	570	624	565	478
$\lambda_{\text{NA,SA}}$	976	1157	1033	920	1024
$\lambda_{\text{AUS,SA}}$	1215	1036	1066	1203	1002
$\lambda_{\text{AS,SA}}$	899	728	1072	757	962
**Joint ESS**					
$\boldsymbol{\Theta}$	1204	1080	1171	1033	1138
$\boldsymbol{\Lambda}$	1337	1190	1451	1285	1148
$(\boldsymbol{\Theta}, \boldsymbol{\Lambda})$	1337	1209	1244	1209	1187

Table S4: Effective sample size estimates for evolutionary parameters for the MRSA analysis.
