# Supplementary material for: Bayesian Inference of Pathogen Phylogeography using the Structured Coalescent Model
Source: PLoS Comput Biol. 2025 Apr 21;21(4):e1012995. doi: 10.1371/journal.pcbi.1012995 (PMC12040344; doi:10.1371/journal.pcbi.1012995)
Supplement: S5 Table — The first column gives the R^ value for the coalescent rate in each deme whilst the remaining columns give the R^ values for backwards-in-time migration rates between pairs of demes. The row gives the source deme for a migration rate and the column gives the target deme (backwards-in-time). The greatest R^ values are highlighted in bold. (PDF) [file pcbi.1012995.s007.pdf]

	θ_x	$\lambda_{x,\text{EUR}}$	$\lambda_{x,\text{NA}}$	$\lambda_{x,\text{AUS}}$	$\lambda_{x,\text{AS}}$	$\lambda_{x,\text{SA}}$
$x = \text{EUR}$	**1.0034**	—	1.0027	1.0029	1.0023	1.0024
$x = \text{NA}$	1.0008	**1.0054**	—	1.0013	1.0025	1.0036
$x = \text{AUS}$	1.0013	1.0012	1.0015	—	1.0012	1.0021
$x = \text{AS}$	1.0005	1.0011	1.0027	1.0013	—	1.0016
$x = \text{SA}$	1.0012	1.0033	1.0026	1.0009	1.0039	—

Table S5: Gelman–Rubin $\hat{R}$ statistics for evolutionary parameters for the MRSA analysis. The first column gives the $\hat{R}$ value for the coalescent rate in each deme whilst the remaining columns give the $\hat{R}$ values for backwards-in-time migration rates between pairs of demes. The row gives the source deme for a migration rate and the column gives the target deme (backwards-in-time). The greatest $\hat{R}$ values are highlighted in **bold**.
