# Supplementary material for: Bayesian Inference of Pathogen Phylogeography using the Structured Coalescent Model
Source: PLoS Comput Biol. 2025 Apr 21;21(4):e1012995. doi: 10.1371/journal.pcbi.1012995 (PMC12040344; doi:10.1371/journal.pcbi.1012995)
Supplement: S2 Fig — (a) Trace plot of the total migration count. The black dashed line indicates the number of required migration events for a maximum parsimony migration history. (b) Stacked trace plot of the proportion of the migration history falling into each deme. (PDF) [file pcbi.1012995.s008.pdf]

(a)

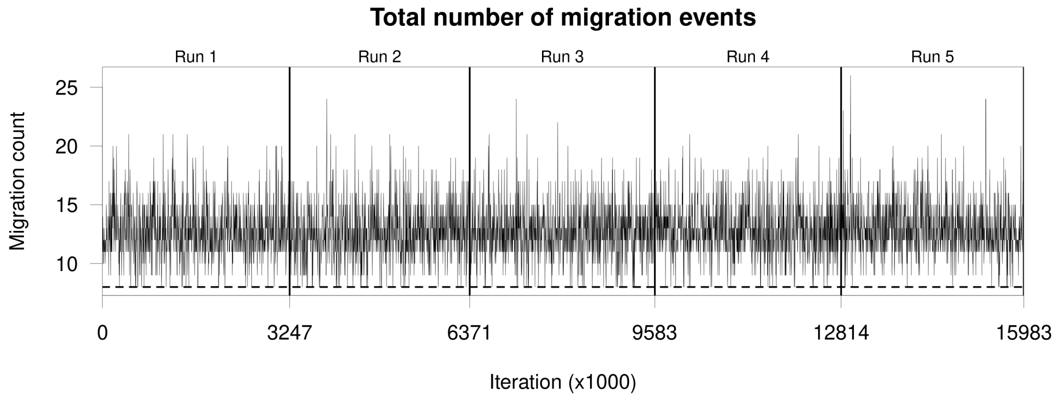

(b)

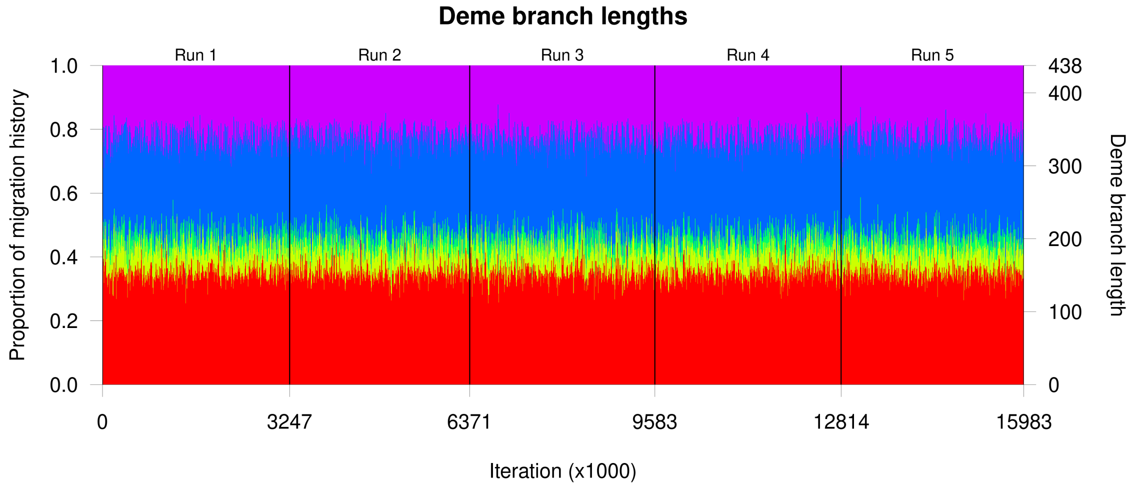

Figure S2: Migration history summary statistics for the MRSA analysis. (a) Trace plot of the total migration count. The black dashed line indicates the number of required migration events for a maximum parsimony migration history. (b) Stacked trace plot of the proportion of the migration history falling into each deme.
