# Supplementary material for: Bayesian Inference of Pathogen Phylogeography using the Structured Coalescent Model
Source: PLoS Comput Biol. 2025 Apr 21;21(4):e1012995. doi: 10.1371/journal.pcbi.1012995 (PMC12040344; doi:10.1371/journal.pcbi.1012995)
Supplement: S3 Fig — The black line indicates the prior density for all migration events. (PDF) [file pcbi.1012995.s009.pdf]

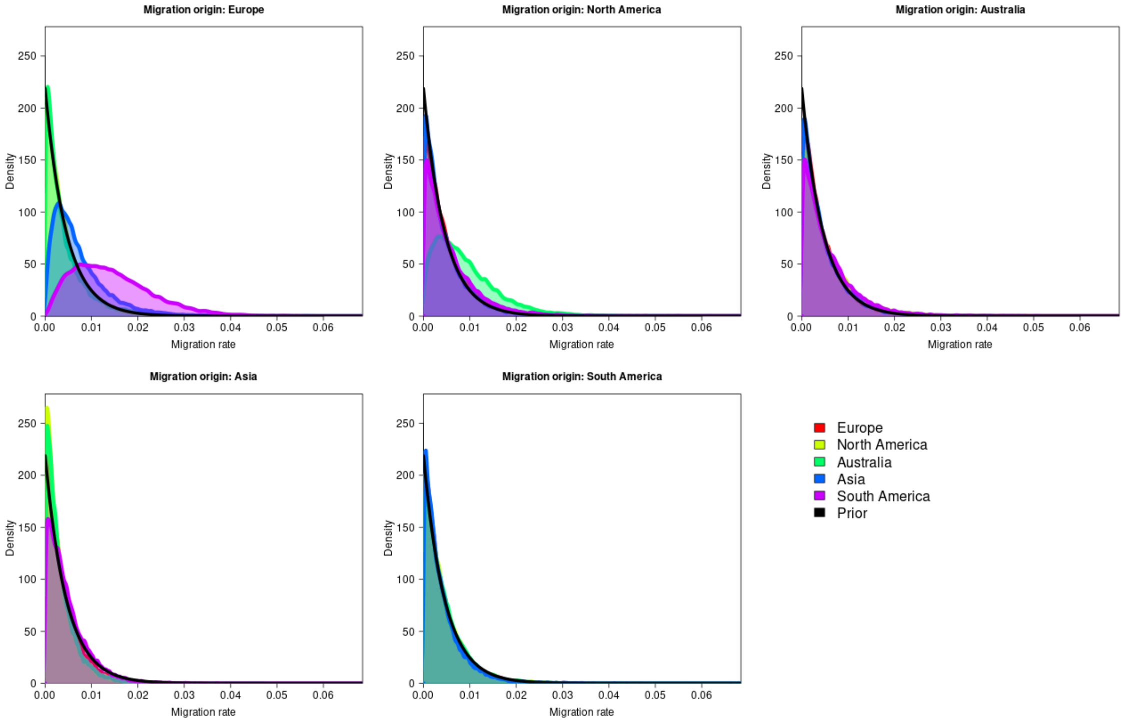

Figure S3: Kernel density estimates of the posterior density of backwards-in-time migration rates, separated by origin deme. The black line indicates the prior density for all migration events.
