# Supplementary material for: Bayesian Inference of Pathogen Phylogeography using the Structured Coalescent Model
Source: PLoS Comput Biol. 2025 Apr 21;21(4):e1012995. doi: 10.1371/journal.pcbi.1012995 (PMC12040344; doi:10.1371/journal.pcbi.1012995)
Supplement: S4 Fig — (a) Log-posterior density evaluations with gamma-distributed priors matching our default priors. (b) Posterior migration counts with gamma-distributed priors matching our default priors. (c) Log-posterior density evaluations with lognormal priors matching the MultiTypeTree default priors. (d) Posterior migration counts with lognormal priors matching the MultiTypeTree default priors. [file pcbi.1012995.s010.pdf]

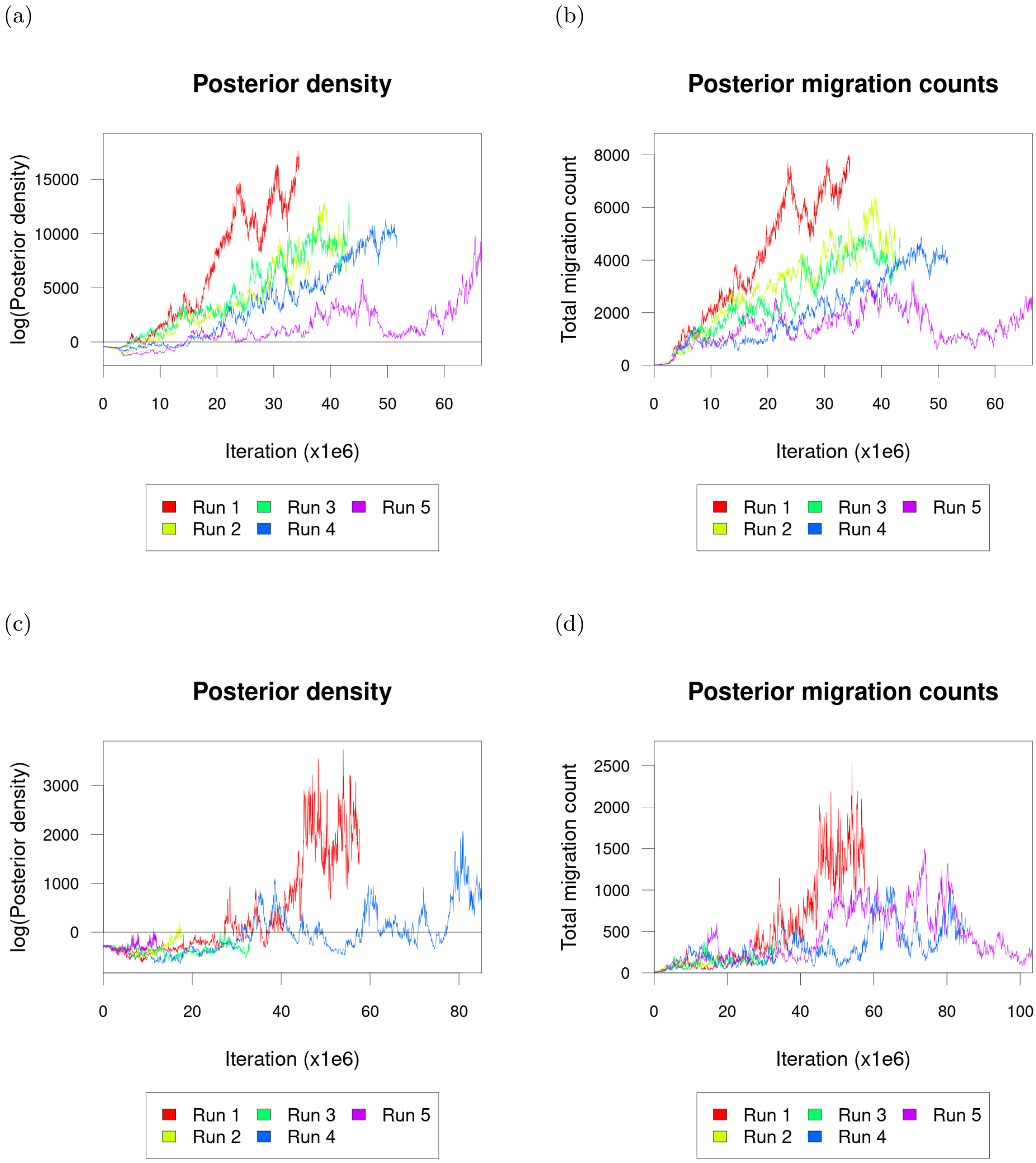

Figure S4: Trace plots for MultiTypeTree MCMC runs. (a) Log-posterior density evaluations with gamma-distributed priors matching our default priors. (b) Posterior migration counts with gamma-distributed priors matching our default priors. (c) Log-posterior density evaluations with lognormal priors matching the MultiTypeTree default priors. (d) Posterior migration counts with lognormal priors matching the MultiTypeTree default priors.
