# Supplementary material for: Bayesian Inference of Pathogen Phylogeography using the Structured Coalescent Model
Source: PLoS Comput Biol. 2025 Apr 21;21(4):e1012995. doi: 10.1371/journal.pcbi.1012995 (PMC12040344; doi:10.1371/journal.pcbi.1012995)
Supplement: S6 Table — (PDF) [file pcbi.1012995.s011.pdf]

	Run 1	Run 2	Run 3	Run 4	Run 5
**Coalescent rates**					
θ_{EUR}	556,145	561,835	559,961	555,628	555,132
θ_{NA}	470,557	514,320	442,479	415,923	335,878
θ_{AUS}	525,612	274,429	447,987	505,616	546,414
θ_{AS}	556,145	557,053	559,744	541,238	558,516
θ_{SA}	508,734	501,516	516,221	501,313	497,908
**Migration rates**					
$\lambda_{\text{NA,EUR}}$	332,529	351,179	333,983	341,559	353,678
$\lambda_{\text{AUS,EUR}}$	330,354	341,070	336,418	331,968	340,394
$\lambda_{\text{AS,EUR}}$	349,361	336,524	346,128	327,882	342,531
$\lambda_{\text{SA,EUR}}$	337,178	349,601	323,434	340,694	324,751
$\lambda_{\text{EUR,NA}}$	294,674	295,940	310,871	296,152	290,205
$\lambda_{\text{AUS,NA}}$	344,220	330,965	347,385	351,365	341,640
$\lambda_{\text{AS,NA}}$	315,578	317,494	331,354	318,554	320,846
$\lambda_{\text{SA,NA}}$	325,335	329,207	327,371	329,794	334,533
$\lambda_{\text{EUR,AUS}}$	273,306	281,617	277,944	266,491	277,345
$\lambda_{\text{NA,AUS}}$	397,104	398,874	395,241	405,865	403,355
$\lambda_{\text{AS,AUS}}$	298,425	308,799	303,121	309,154	301,516
$\lambda_{\text{SA,AUS}}$	334,089	338,532	333,949	339,953	340,454
$\lambda_{\text{EUR,AS}}$	387,860	387,454	392,104	398,614	378,268
$\lambda_{\text{NA,AS}}$	341,455	337,976	331,630	345,753	345,102
$\lambda_{\text{AUS,AS}}$	331,220	344,283	330,497	326,052	338,404
$\lambda_{\text{SA,AS}}$	339,531	332,061	340,037	336,006	331,895
$\lambda_{\text{EUR,SA}}$	373,273	397,009	386,785	403,733	385,839
$\lambda_{\text{NA,SA}}$	344,015	335,439	336,643	333,193	334,575
$\lambda_{\text{AUS,SA}}$	335,397	339,245	329,280	334,695	345,597
$\lambda_{\text{AS,SA}}$	334,579	346,589	357,696	344,439	347,007

Table S6: ESS for the MASCOT analysis of the MRSA dataset.
