# Supplementary material for: Bayesian Inference of Pathogen Phylogeography using the Structured Coalescent Model
Source: PLoS Comput Biol. 2025 Apr 21;21(4):e1012995. doi: 10.1371/journal.pcbi.1012995 (PMC12040344; doi:10.1371/journal.pcbi.1012995)
Supplement: S7 Table — Greatest R^ values are highlighted in bold. (PDF) [file pcbi.1012995.s012.pdf]

	θ_x	$\lambda_{x,\mathrm{EUR}}$	$\lambda_{x,\mathrm{NA}}$	$\lambda_{x,\mathrm{AUS}}$	$\lambda_{x,\mathrm{AS}}$	$\lambda_{x,\mathrm{SA}}$
$x = \mathrm{EUR}$	1.0000	—	1.0001	1.0004	1.0001	0.9999
$x = \mathrm{NA}$	**1.0003**	1.0006	—	1.0000	1.0000	1.0004
$x = \mathrm{AUS}$	0.9999	1.0000	1.0002	—	1.0003	1.0002
$x = \mathrm{AS}$	0.9999	1.0004	1.0005	1.0002	—	1.0002
$x = \mathrm{SA}$	1.0001	1.0000	1.0000	1.0002	**1.0010**	—

Table S7: Gelman–Rubin $\hat{R}$ statistics for the MASCOT analysis of the MRSA dataset. Greatest $\hat{R}$ values are highlighted in **bold**.
