# Supplementary material for: Bayesian Inference of Pathogen Phylogeography using the Structured Coalescent Model
Source: PLoS Comput Biol. 2025 Apr 21;21(4):e1012995. doi: 10.1371/journal.pcbi.1012995 (PMC12040344; doi:10.1371/journal.pcbi.1012995)
Supplement: S5 Fig — (a) Trace plot of the total migration count. The black dashed line indicates the number of required migration events for a maximum parsimony migration history. (b) Stacked trace plot of the proportion of the migration history falling into each deme across the five MCMC samples. (PDF) [file pcbi.1012995.s013.pdf]

(a)

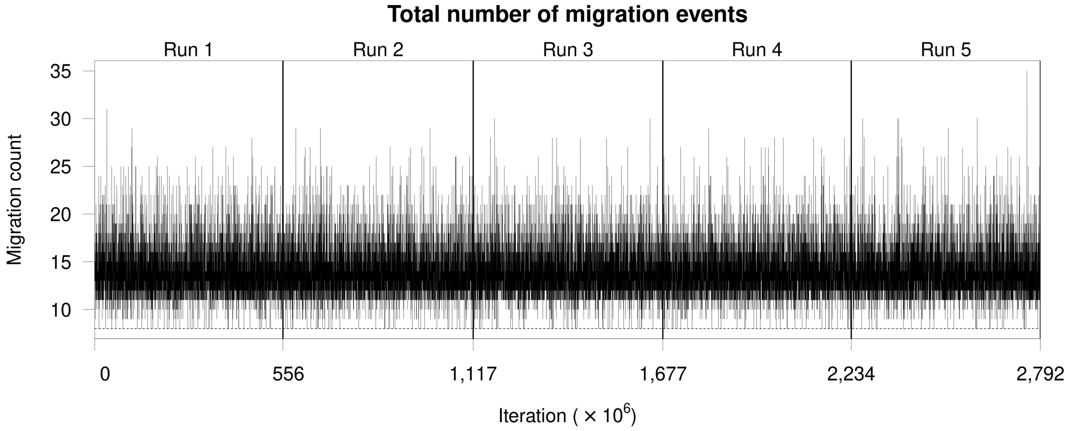

(b)

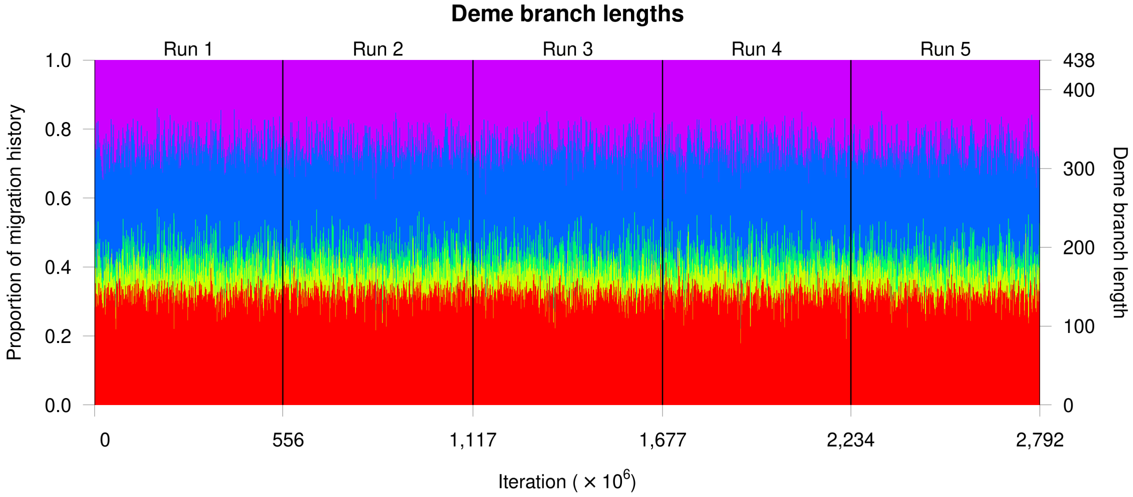

Figure S5: Trace plots of migration history summary statistics for the MASCOT analysis of the MRSA dataset. (a) Trace plot of the total migration count. The black dashed line indicates the number of required migration events for a maximum parsimony migration history. (b) Stacked trace plot of the proportion of the migration history falling into each deme across the five MCMC samples.
