# Supplementary material for: Bayesian Inference of Pathogen Phylogeography using the Structured Coalescent Model
Source: PLoS Comput Biol. 2025 Apr 21;21(4):e1012995. doi: 10.1371/journal.pcbi.1012995 (PMC12040344; doi:10.1371/journal.pcbi.1012995)
Supplement: S6 Fig — (PDF) [file pcbi.1012995.s014.pdf]

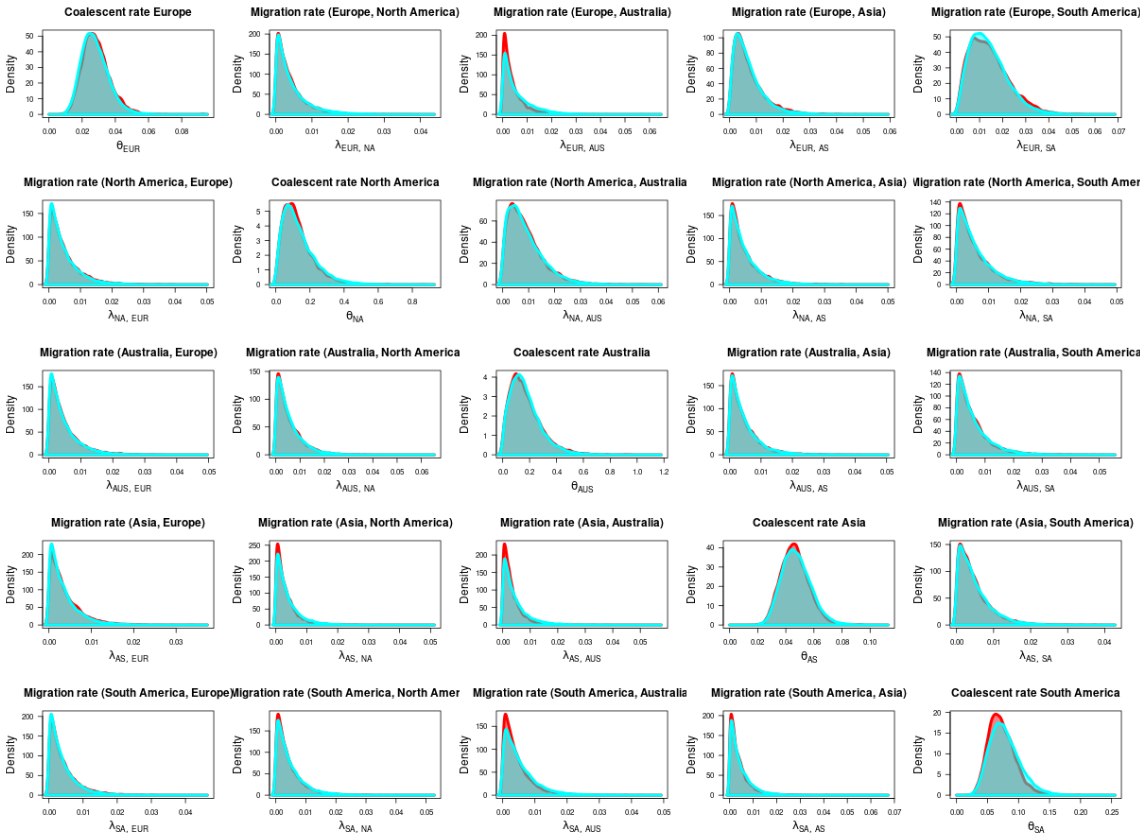

Figure S6: Empirical posterior density estimates for evolutionary parameters sampled using Local DTA analysis (shown in red) and MASCOT (shown in blue).
