# Supplementary material for: Bayesian Inference of Pathogen Phylogeography using the Structured Coalescent Model
Source: PLoS Comput Biol. 2025 Apr 21;21(4):e1012995. doi: 10.1371/journal.pcbi.1012995 (PMC12040344; doi:10.1371/journal.pcbi.1012995)
Supplement: S8 Table — (PDF) [file pcbi.1012995.s016.pdf]

	Default priors					Exp(1) priors				
	Run 1	Run 2	Run 3	Run 4	Run 5	Run 1	Run 2	Run 3	Run 4	Run 5
**Coalescent rates**										
θ_{ANS}	928	1122	790	1660	1001	252	297	203	231	192
θ_{CHA}	971	1510	1412	1101	1673	253	685	713	586	469
θ_{GAL}	980	1279	1334	1251	1280	195	267	162	132	110
θ_{PAS}	1085	1702	1083	1460	1541	268	90	138	218	252
θ_{MEX}	1482	1418	1499	1039	1186	43	137	41	60	130
**Migration rates**										
$\lambda_{\mathrm{CHA,ANS}}$	720	876	1002	910	866	119	368	356	232	169
$\lambda_{\mathrm{GAL,ANS}}$	983	1335	1045	1355	1079	78	287	265	260	187
$\lambda_{\mathrm{PAS,ANS}}$	832	735	1032	728	800	129	156	253	218	99
$\lambda_{\mathrm{MEX,ANS}}$	802	886	1092	804	868	146	99	176	126	87
$\lambda_{\mathrm{ANS,CHA}}$	287	1185	1059	606	1166	34	109	111	72	199
$\lambda_{\mathrm{GAL,CHA}}$	1733	1371	1413	1595	1541	72	104	87	70	57
$\lambda_{\mathrm{PAS,CHA}}$	1545	1454	1695	1345	1541	94	49	184	131	79
$\lambda_{\mathrm{MEX,CHA}}$	1234	1106	1687	1198	1383	85	57	97	86	86
$\lambda_{\mathrm{ANS,GAL}}$	847	826	976	835	866	109	97	44	48	50
$\lambda_{\mathrm{CHA,GAL}}$	1008	988	1012	802	927	82	199	149	107	110
$\lambda_{\mathrm{PAS,GAL}}$	1196	1335	1269	1555	1292	98	41	152	78	70
$\lambda_{\mathrm{MEX,GAL}}$	1620	1662	971	1128	1399	77	54	37	54	90
$\lambda_{\mathrm{ANS,PAS}}$	625	668	838	666	266	99	67	28	55	125
$\lambda_{\mathrm{CHA,PAS}}$	901	1121	1137	673	1155	65	72	82	145	108
$\lambda_{\mathrm{GAL,PAS}}$	1216	1459	1677	1766	1262	121	146	127	95	54
$\lambda_{\mathrm{MEX,PAS}}$	1602	1262	1053	1400	1632	76	94	105	46	70
$\lambda_{\mathrm{ANS,MEX}}$	648	655	565	507	631	162	82	41	36	71
$\lambda_{\mathrm{CHA,MEX}}$	847	666	717	596	834	74	248	190	125	205
$\lambda_{\mathrm{GAL,MEX}}$	1149	1494	1061	1240	1118	74	141	81	52	111
$\lambda_{\mathrm{PAS,MEX}}$	1447	1459	1363	1204	1134	107	61	134	90	115

Table S8: Effective sample size estimates for evolutionary for the AIV analysis with default gamma-distributed priors (left) and Exp(1) priors (right).
