# Supplementary material for: Bayesian Inference of Pathogen Phylogeography using the Structured Coalescent Model
Source: PLoS Comput Biol. 2025 Apr 21;21(4):e1012995. doi: 10.1371/journal.pcbi.1012995 (PMC12040344; doi:10.1371/journal.pcbi.1012995)
Supplement: S9 Table — The first column gives the R^ value for the coalescent rate in each deme whilst the remaining columns give the R^ values for backwards-in-time migration rates between pairs of demes. The row gives the source deme for a migration rate and the column gives the target deme (backwards-in-time). The greatest R^ values are highlighted in bold. (PDF) [file pcbi.1012995.s017.pdf]

(a)

	θ_x	$\lambda_{x,\text{ANS}}$	$\lambda_{x,\text{CHA}}$	$\lambda_{x,\text{GAL}}$	$\lambda_{x,\text{PAS}}$	$\lambda_{x,\text{MEX}}$
$x = \text{ANS}$	1.0016	—	**1.0209**	1.0034	1.0120	1.0083
$x = \text{CHA}$	1.0014	1.0005	—	1.0016	1.0056	1.0060
$x = \text{GAL}$	1.0021	1.0013	1.0010	—	1.0022	1.0010
$x = \text{PAS}$	1.0015	1.0023	1.0017	1.0035	—	1.0017
$x = \text{MEX}$	**1.0031**	1.0014	1.0021	1.0005	1.0013	—

(b)

	θ_x	$\lambda_{x,\text{ANS}}$	$\lambda_{x,\text{CHA}}$	$\lambda_{x,\text{GAL}}$	$\lambda_{x,\text{PAS}}$	$\lambda_{x,\text{MEX}}$
$x = \text{ANS}$	1.2681	—	2.2809	1.2762	1.8498	**3.6655**
$x = \text{CHA}$	1.1470	1.0708	—	1.0141	1.8452	1.3401
$x = \text{GAL}$	1.0772	1.2158	1.1500	—	1.1119	1.4620
$x = \text{PAS}$	**2.2424**	1.1923	1.2051	1.0745	—	1.2278
$x = \text{MEX}$	1.8165	1.4964	1.2981	1.5730	1.8347	—

Table S9: Gelman–Rubin $\hat{R}$ statistics for evolutionary parameters for the AIV analysis with (a) default priors; (b) Exp(1) priors. The first column gives the $\hat{R}$ value for the coalescent rate in each deme whilst the remaining columns give the $\hat{R}$ values for backwards-in-time migration rates between pairs of demes. The row gives the source deme for a migration rate and the column gives the target deme (backwards-in-time). The greatest $\hat{R}$ values are highlighted in **bold**.
