# Supplementary material for: Bayesian Inference of Pathogen Phylogeography using the Structured Coalescent Model
Source: PLoS Comput Biol. 2025 Apr 21;21(4):e1012995. doi: 10.1371/journal.pcbi.1012995 (PMC12040344; doi:10.1371/journal.pcbi.1012995)
Supplement: S11 Fig — (a) Trace plot of the total migration count. The black dashed line indicates the number of required migration events (M = 37) for a maximum parsimony migration history. (b) Stacked trace plot of the proportion of the migration history falling into each deme. (PDF) [file pcbi.1012995.s021.pdf]

(a)

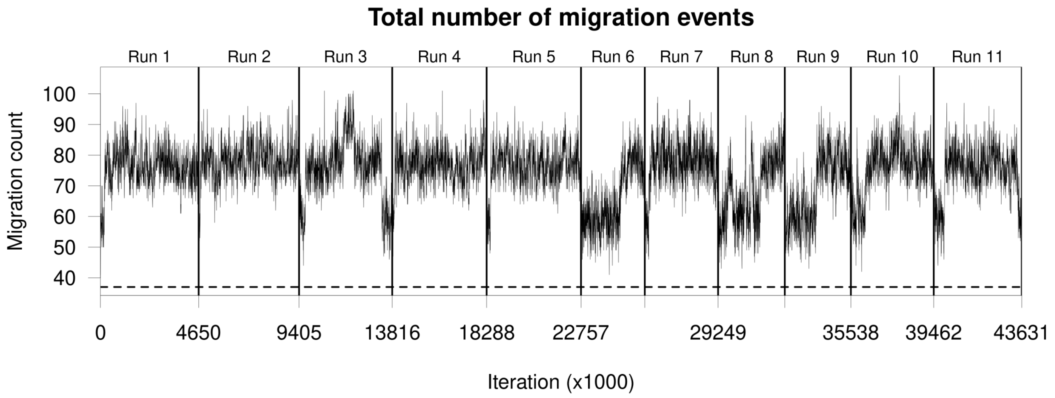

(b)

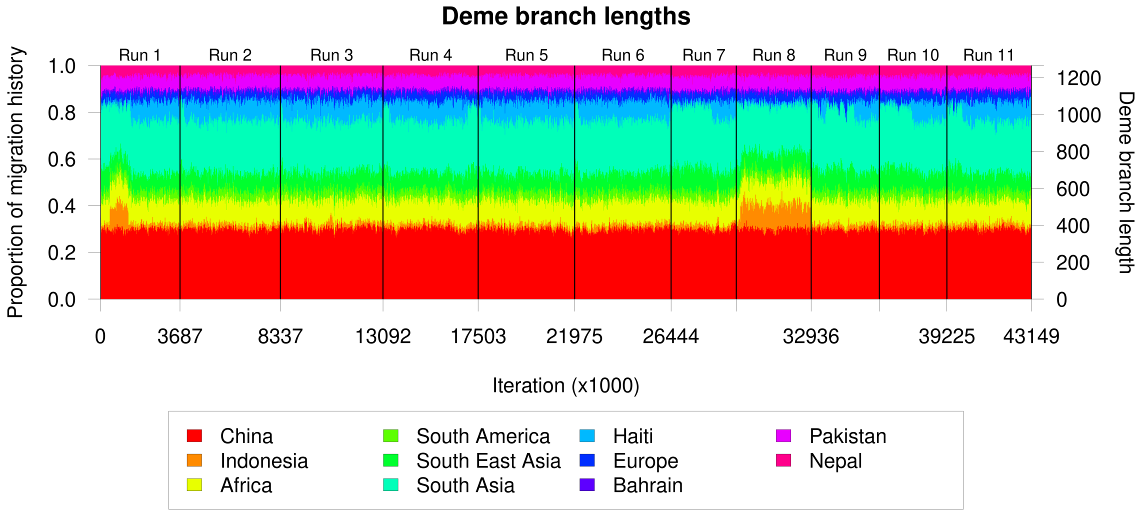

Figure S11: Migration history summary statistics for the Cholera analysis with default priors. (a) Trace plot of the total migration count. The black dashed line indicates the number of required migration events ($M = 37$) for a maximum parsimony migration history. (b) Stacked trace plot of the proportion of the migration history falling into each deme.
