# Supplementary material for: Bayesian Inference of Pathogen Phylogeography using the Structured Coalescent Model
Source: PLoS Comput Biol. 2025 Apr 21;21(4):e1012995. doi: 10.1371/journal.pcbi.1012995 (PMC12040344; doi:10.1371/journal.pcbi.1012995)
Supplement: S12 Fig — (PDF) [file pcbi.1012995.s022.pdf]

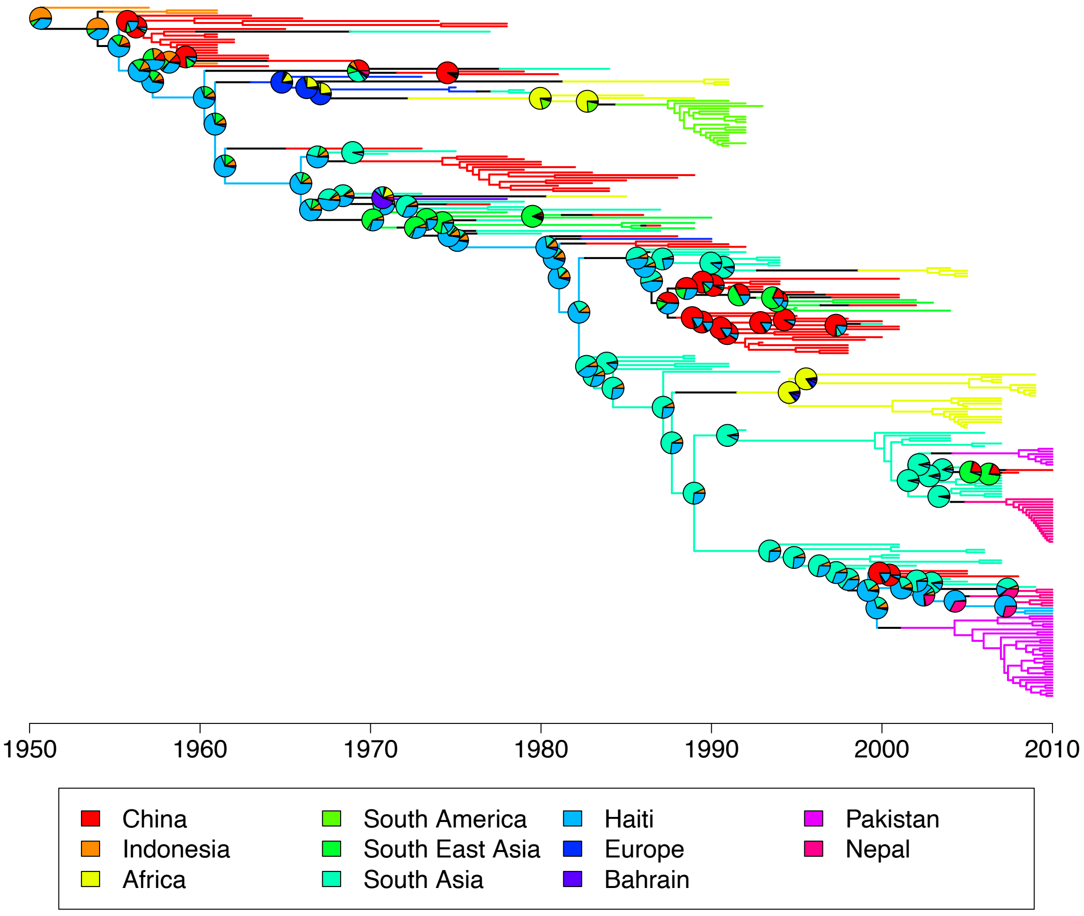

Figure S12: 60% consensus migration history for the preliminary Cholera analysis using default priors.
