# Supplementary material for: Bayesian Inference of Pathogen Phylogeography using the Structured Coalescent Model
Source: PLoS Comput Biol. 2025 Apr 21;21(4):e1012995. doi: 10.1371/journal.pcbi.1012995 (PMC12040344; doi:10.1371/journal.pcbi.1012995)
Supplement: S10 Table [file pcbi.1012995.s023.pdf]

	Run 1	Run 2	Run 3	Run 4	Run 5	Run 6	Run 7	Run 8	Run 9	Run 10	Run 11
**Coalescent rates**											
θ_{CHN}	863	1131	1275	1381	1402	1074	**715**	737	842	1125	1021
θ_{IDN}	**307**	1376	1201	1002	1145	1178	948	883	996	1218	1291
θ_{AFR}	601	781	674	609	656	763	659	**520**	716	574	801
θ_{SA}	1931	1947	2630	1895	2024	1959	1378	1504	**1001**	2133	1707
θ_{IC}	587	811	894	564	814	884	419	**339**	459	569	487
θ_{SAS}	745	705	1050	478	789	1000	519	**436**	453	706	895
θ_{HTI}	98	182	205	148	213	182	**88**	92	112	124	139
θ_{EUR}	971	1067	993	718	921	1274	578	**301**	433	414	634
θ_{BHR}	1150	1500	1299	1678	1878	1784	1487	1052	1351	**876**	1347
θ_{PAK}	1915	2470	2163	2035	2146	2404	**1373**	1545	1663	2458	2324
θ_{NPL}	1424	2130	2145	1781	2070	1789	1249	1250	**1032**	1516	1428
**Coalescent rates**											
$\lambda_{\text{IDN,CHN}}$	1110	1287	1149	1661	1366	1247	971	990	**921**	1013	1186
$\lambda_{\text{AFR,CHN}}$	1624	1505	1811	2293	1279	1723	1092	1383	**1014**	1533	1791
$\lambda_{\text{SA,CHN}}$	1554	2039	1882	2211	1572	1461	1337	**1249**	1454	1306	1594
$\lambda_{\text{IC,CHN}}$	819	841	799	851	819	767	534	**469**	611	569	651
$\lambda_{\text{SAS,CHN}}$	835	754	978	633	818	649	684	534	565	601	**518**
$\lambda_{\text{HTI,CHN}}$	1160	1975	1582	1644	1670	1249	961	1142	**872**	1129	1202
$\lambda_{\text{EUR,CHN}}$	1357	1611	1155	1426	2105	1553	**826**	1393	942	1440	1399
$\lambda_{\text{BHR,CHN}}$	1790	2273	2365	1801	1672	1719	1538	1653	1521	1705	**1477**
$\lambda_{\text{PAK,CHN}}$	1408	1874	1740	1717	1709	1752	1076	1286	**902**	1996	1641
$\lambda_{\text{NPL,CHN}}$	1297	1719	1709	1580	1617	1116	**928**	1148	1293	1551	1457
$\lambda_{\text{CHN,IDN}}$	**39**	701	621	819	270	456	383	316	343	387	693
$\lambda_{\text{AFR,IDN}}$	634	587	**360**	636	970	741	483	570	625	587	1030
$\lambda_{\text{SA,IDN}}$	1350	3259	1819	2042	2005	1877	1303	1869	**1178**	2278	1336
$\lambda_{\text{IC,IDN}}$	**163**	1206	1077	1585	997	1162	750	966	1162	718	775
$\lambda_{\text{SAS,IDN}}$	**27**	1262	1266	922	978	724	677	590	781	937	933
$\lambda_{\text{HTI,IDN}}$	**752**	1211	1231	918	834	951	944	1290	956	1020	1129
$\lambda_{\text{EUR,IDN}}$	**392**	1327	1412	1238	1443	1299	695	873	1019	1584	1629
$\lambda_{\text{BHR,IDN}}$	**938**	1959	1838	1587	2223	1581	953	1719	1173	1814	1690
$\lambda_{\text{PAK,IDN}}$	**671**	1862	1770	1330	1763	1654	1106	1817	1325	2348	1698
$\lambda_{\text{NPL,IDN}}$	**516**	1835	2052	2030	1938	1423	1017	1185	1102	1314	2181
$\lambda_{\text{CHN,AFR}}$	1539	1684	1615	1726	1698	1679	1235	1452	**1102**	1477	1441
$\lambda_{\text{IDN,AFR}}$	1486	2006	1928	1703	1765	1922	**1015**	1147	1378	2004	1383
$\lambda_{\text{SA,AFR}}$	1200	1797	1413	1588	1271	1479	1157	1190	**909**	1226	1187
$\lambda_{\text{IC,AFR}}$	1670	1864	1847	1678	1951	2446	1331	**1254**	1680	1778	1292
$\lambda_{\text{SAS,AFR}}$	1672	1709	2599	1807	1890	1562	**985**	1626	1481	1621	1731
$\lambda_{\text{HTI,AFR}}$	1501	1982	2233	1698	2309	1999	**834**	1456	1025	1447	1771
$\lambda_{\text{EUR,AFR}}$	637	895	839	777	917	598	**510**	608	577	660	875
$\lambda_{\text{BHR,AFR}}$	1233	1607	1363	1490	1264	1318	**1071**	1089	**1071**	1348	1880
$\lambda_{\text{PAK,AFR}}$	1859	1630	2547	1824	2008	1817	1628	**1289**	1362	1578	1742
$\lambda_{\text{NPL,AFR}}$	1794	1910	2183	1905	2046	2060	**1165**	1527	1657	1427	1884
$\lambda_{\text{CHN,SA}}$	1177	2349	2236	1755	1538	1814	1371	1322	1117	1084	**805**
$\lambda_{\text{IDN,SA}}$	**1139**	2052	1845	2183	1838	1776	1411	1595	1252	1606	1634
$\lambda_{\text{AFR,SA}}$	854	661	731	709	631	505	**450**	534	476	495	626
$\lambda_{\text{IC,SA}}$	1853	2046	2194	1897	1883	2525	1712	1250	1246	1331	**1141**
$\lambda_{\text{SAS,SA}}$	998	2042	1840	1967	1616	1153	**782**	1121	1038	1286	1781
$\lambda_{\text{HTI,SA}}$	1784	1845	2107	2195	2036	1781	1312	**1202**	1413	1745	1563
$\lambda_{\text{EUR,SA}}$	1205	1546	1021	1201	1428	1015	**917**	1141	1135	949	1601
$\lambda_{\text{BHR,SA}}$	1792	2066	1765	2275	1931	1711	1597	1349	**1257**	2116	1671
$\lambda_{\text{PAK,SA}}$	1577	1836	2354	1640	1969	2148	1807	1435	**1394**	2328	2227
$\lambda_{\text{NPL,SA}}$	1862	2112	1743	1662	1446	1740	**1221**	1492	1593	1500	1772
$\lambda_{\text{CHN,IC}}$	159	246	267	211	238	258	**136**	147	177	182	180
$\lambda_{\text{IDN,IC}}$	480	629	781	680	932	900	363	316	**296**	589	547
$\lambda_{\text{AFR,IC}}$	1541	2091	1608	1775	1430	1492	1289	**1175**	1601	1445	1779
$\lambda_{\text{SA,IC}}$	1550	2290	2090	2062	1331	1479	**942**	1166	1371	2219	2007
$\lambda_{\text{SAS,IC}}$	311	703	663	324	700	430	194	**191**	224	278	250
$\lambda_{\text{HTI,IC}}$	763	783	630	781	657	923	740	709	618	756	**558**
$\lambda_{\text{EUR,IC}}$	1169	1690	1882	833	1347	1336	**525**	668	946	756	843
$\lambda_{\text{BHR,IC}}$	1565	2349	1662	1879	1698	1566	948	1436	**746**	1581	1423
$\lambda_{\text{PAK,IC}}$	1294	2305	1766	2339	1807	2411	**1162**	1368	1347	1651	1897
$\lambda_{\text{NPL,IC}}$	1195	1632	2148	1513	1266	1575	**1100**	1707	1251	1457	1473
$\lambda_{\text{CHN,SAS}}$	216	267	344	221	294	260	**150**	181	155	225	221
$\lambda_{\text{IDN,SAS}}$	**617**	1074	1072	1169	1397	1676	890	1311	1095	994	1571
$\lambda_{\text{AFR,SAS}}$	962	972	1140	820	851	973	**614**	706	722	675	1121
$\lambda_{\text{SA,SAS}}$	1601	1775	2005	1783	1709	1755	**1217**	1275	1427	1698	1837
$\lambda_{\text{IC,SAS}}$	575	597	806	633	762	835	**367**	516	476	486	595
$\lambda_{\text{HTI,SAS}}$	754	601	540	598	654	621	692	667	**520**	541	955

$\lambda_{EUR,SAS}$	877	1055	1172	686	1035	800	534	**476**	553	644	895
$\lambda_{BHR,SAS}$	991	1064	1378	1014	1333	1146	837	856	**828**	1044	839
$\lambda_{PAK,SAS}$	913	1210	1704	837	1254	1636	**568**	596	670	939	735
$\lambda_{NPL,SAS}$	**604**	1088	1214	969	1102	1157	805	721	800	757	1179
$\lambda_{CHN,HTI}$	51	126	176	90	131	118	**34**	43	43	83	83
$\lambda_{IDN,HTI}$	207	445	462	341	417	353	211	195	**179**	297	306
$\lambda_{AFR,HTI}$	677	826	735	822	739	644	**479**	707	494	761	947
$\lambda_{SA,HTI}$	1133	1517	1315	1126	1272	1336	902	1457	1223	995	**810**
$\lambda_{IC,HTI}$	168	393	428	259	413	372	**157**	167	170	221	254
$\lambda_{SAS,HTI}$	52	135	216	56	149	144	31	**28**	30	62	104
$\lambda_{EUR,HTI}$	344	918	706	437	718	670	299	**287**	331	415	522
$\lambda_{BHR,HTI}$	624	804	777	1006	904	794	**363**	415	689	487	826
$\lambda_{PAK,HTI}$	710	1096	1039	751	968	929	629	508	**385**	725	684
$\lambda_{NPL,HTI}$	246	599	844	351	533	586	227	**206**	235	358	432
$\lambda_{CHN,EUR}$	1177	1539	1208	375	1185	1367	202	**55**	121	91	946
$\lambda_{IDN,EUR}$	1324	1610	1892	1268	1868	1472	913	**530**	897	712	1435
$\lambda_{AFR,EUR}$	502	587	519	**290**	438	575	466	391	397	314	509
$\lambda_{SA,EUR}$	1127	1674	1324	1361	1173	1484	1395	**929**	995	1239	1309
$\lambda_{IC,EUR}$	1360	2058	2463	1863	1941	2164	899	**573**	615	1146	1527
$\lambda_{SAS,EUR}$	984	1789	1331	755	1946	1360	249	**62**	314	174	823
$\lambda_{HTI,EUR}$	1228	2225	2051	1354	1789	1672	1465	981	**871**	1184	1538
$\lambda_{BHR,EUR}$	1538	1710	1619	2009	2204	1863	1379	959	**934**	1671	2056
$\lambda_{PAK,EUR}$	1773	1788	1883	1499	1606	2065	1140	**978**	1425	1419	1280
$\lambda_{NPL,EUR}$	1334	1568	2312	2070	1609	1530	1009	**315**	811	558	1130
$\lambda_{CHN,BHR}$	1006	1016	1333	1200	1333	1205	**593**	848	947	915	1230
$\lambda_{IDN,BHR}$	1528	1816	1825	2453	2027	1844	**1257**	1612	1736	2040	1695
$\lambda_{AFR,BHR}$	**395**	730	613	826	602	548	592	431	495	430	447
$\lambda_{SA,BHR}$	1719	2409	2030	2461	1764	1739	1392	1398	**1262**	2274	1710
$\lambda_{IC,BHR}$	**1224**	1851	2092	1504	1509	1374	1335	1273	1321	1536	1669
$\lambda_{SAS,BHR}$	809	1277	1057	1645	1628	1354	**682**	1092	1097	743	1399
$\lambda_{HTI,BHR}$	1670	2116	2921	2173	1728	1724	**931**	1251	1110	1486	1433
$\lambda_{EUR,BHR}$	1426	1812	1448	1178	1232	1165	**955**	1139	1139	1639	1366
$\lambda_{PAK,BHR}$	1536	1971	1559	2091	1923	1863	**1037**	1164	1185	1518	1794
$\lambda_{NPL,BHR}$	**1111**	1543	2582	1872	2894	1848	1528	1217	1181	1437	1227
$\lambda_{CHN,PAK}$	1172	1966	1260	1471	1275	1189	917	1047	**607**	1100	1267
$\lambda_{IDN,PAK}$	**1058**	2578	2160	1964	2168	1532	1148	1488	1246	1935	1579
$\lambda_{AFR,PAK}$	916	1337	1180	1161	813	1203	1076	1013	**787**	1510	1240
$\lambda_{SA,PAK}$	1352	2757	1999	2124	1873	1607	2259	**1292**	1718	2010	2382
$\lambda_{IC,PAK}$	1894	1784	1748	1674	1772	1432	1539	**1082**	1085	1365	1589
$\lambda_{SAS,PAK}$	1236	1434	1389	1126	1353	1038	1026	909	**741**	1389	855
$\lambda_{HTI,PAK}$	1371	1722	2679	1845	2148	1836	1426	1335	**916**	2189	1854
$\lambda_{EUR,PAK}$	1829	2071	1295	1622	1738	1450	**953**	1448	1051	1493	1806
$\lambda_{BHR,PAK}$	1574	1668	1881	1796	2137	2035	**1217**	1359	1609	1411	1821
$\lambda_{NPL,PAK}$	1894	1906	2236	2155	1462	1989	1232	1163	**1092**	2164	1858
$\lambda_{CHN,NPL}$	1172	856	1066	953	780	835	958	**632**	738	812	1072
$\lambda_{IDN,NPL}$	1345	1794	1921	2015	2079	1715	**1170**	1583	1449	1399	1586
$\lambda_{AFR,NPL}$	738	760	**573**	1036	786	1041	838	632	1074	1023	1128
$\lambda_{SA,NPL}$	1928	2379	2119	1620	1680	1858	1541	1266	**975**	1835	1576
$\lambda_{IC,NPL}$	1134	1126	1436	1162	1474	964	1045	1453	**837**	1157	1416
$\lambda_{SAS,NPL}$	567	687	686	857	551	612	634	526	**459**	547	718
$\lambda_{HTI,NPL}$	533	939	1268	766	1018	994	**465**	495	522	601	720
$\lambda_{EUR,NPL}$	1496	1488	1440	1178	967	1058	1042	**811**	899	1335	1092
$\lambda_{BHR,NPL}$	1388	1808	2163	2190	1846	2288	1586	**1033**	1091	1485	1792
$\lambda_{PAK,NPL}$	1334	1884	2427	1954	1759	1533	1322	1572	**1060**	2069	1753

Table S10: Effective sample size estimates for evolutionary parameters for the cholera analysis.
