# Supplementary material for: Bayesian Inference of Pathogen Phylogeography using the Structured Coalescent Model
Source: PLoS Comput Biol. 2025 Apr 21;21(4):e1012995. doi: 10.1371/journal.pcbi.1012995 (PMC12040344; doi:10.1371/journal.pcbi.1012995)
Supplement: S11 Table — The first column gives the R^ value for the coalescent rate in each deme whilst the remaining columns give the R^ values for backwards-in-time migration rates between pairs of demes. The row gives the source deme for a migration rate and the column gives the target deme (backwards-in-time). (PDF) [file pcbi.1012995.s024.pdf]

	θ_x	$\lambda_{x,\mathrm{CHN}}$	$\lambda_{x,\mathrm{IDN}}$	$\lambda_{x,\mathrm{AFR}}$	$\lambda_{x,\mathrm{SA}}$	$\lambda_{x,\mathrm{SEA}}$	$\lambda_{x,\mathrm{SAS}}$	$\lambda_{x,\mathrm{HTI}}$	$\lambda_{x,\mathrm{EUR}}$	$\lambda_{x,\mathrm{BHR}}$	$\lambda_{x,\mathrm{PAK}}$	$\lambda_{x,\mathrm{NPL}}$
$x = \mathrm{CHN}$	1.0012	—	1.0011	1.0004	1.0005	1.0016	1.0006	1.0275	1.0010	1.0005	1.0007	1.0002
$x = \mathrm{IDN}$	1.0004	1.0004	—	1.0005	1.0002	1.0012	1.0008	1.0001	1.0003	1.0003	1.0002	1.0005
$x = \mathrm{AFR}$	1.0004	1.0005	1.0005	—	1.0006	1.0006	1.0002	1.0004	1.0010	1.0005	1.0004	1.0004
$x = \mathrm{SA}$	1.0001	1.0003	1.0003	1.0003	—	1.0003	1.0002	1.0003	1.0004	1.0003	1.0004	1.0004
$x = \mathrm{SEA}$	1.0005	1.0003	1.0008	1.0003	1.0005	—	1.0007	1.0016	1.0007	1.0003	1.0002	1.0006
$x = \mathrm{SAS}$	1.0004	1.0004	1.0011	1.0002	1.0005	1.0010	—	1.0142	1.0006	1.0008	1.0005	1.0004
$x = \mathrm{HTI}$	1.0007	1.0006	1.0003	1.0004	1.0004	1.0002	1.0003	—	1.0003	1.0003	1.0003	1.0005
$x = \mathrm{EUR}$	1.0005	1.0005	1.0003	1.0008	1.0004	1.0002	1.0002	1.0005	—	1.0003	1.0002	1.0002
$x = \mathrm{BHR}$	1.0005	1.0003	1.0002	1.0003	1.0002	1.0002	1.0003	1.0005	1.0002	—	1.0002	1.0000
$x = \mathrm{PAK}$	1.0002	1.0002	1.0004	1.0004	1.0005	1.0005	1.0003	1.0005	1.0003	1.0003	—	1.0002
$x = \mathrm{NPL}$	1.0003	1.0006	1.0002	1.0004	1.0004	1.0004	1.0002	1.0006	1.0005	1.0002	1.0002	—

Table S11: Gelman–Rubin $\hat{R}$ statistics for evolutionary parameters for the cholera analysis. The first column gives the $\hat{R}$ value for the coalescent rate in each deme whilst the remaining columns give the $\hat{R}$ values for backwards-in-time migration rates between pairs of demes. The row gives the source deme for a migration rate and the column gives the target deme (backwards-in-time).
