# Supplementary material for: Bayesian Inference of Pathogen Phylogeography using the Structured Coalescent Model
Source: PLoS Comput Biol. 2025 Apr 21;21(4):e1012995. doi: 10.1371/journal.pcbi.1012995 (PMC12040344; doi:10.1371/journal.pcbi.1012995)
Supplement: S15 Fig — The width of each arrow denotes the relative frequency with which that migration type was observed. (PDF) [file pcbi.1012995.s027.pdf]

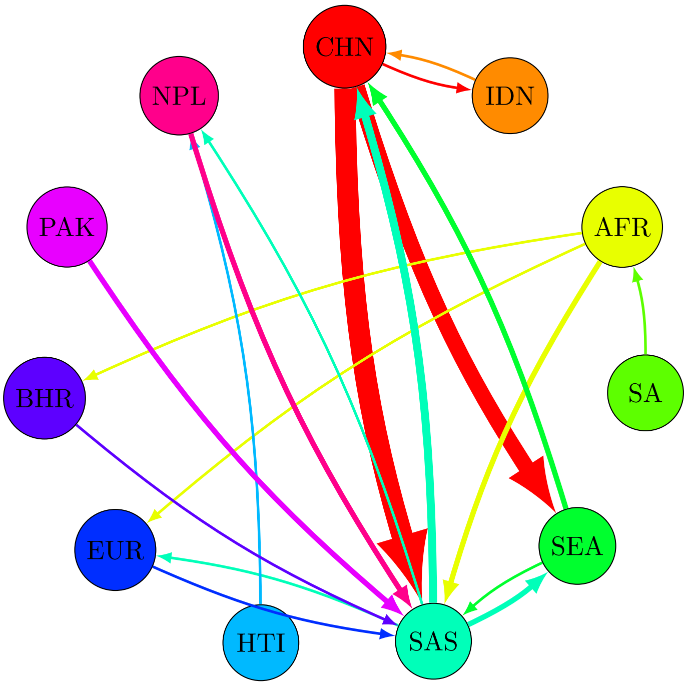

Figure S15: Median number of **backwards-in-time** migration events between pairs of demes in the cholera analysis. The width of each arrow denotes the relative frequency with which that migration type was observed.
